# Synthesis and Antiproliferative Insights of Lipophilic Ru(II)-Hydroxy Stearic Acid Hybrid Species

**DOI:** 10.3390/molecules28104051

**Published:** 2023-05-12

**Authors:** Giacomo Drius, Silvia Bordoni, Carla Boga, Magda Monari, Jessica Fiori, Erika Esposito, Chiara Zalambani, Luca Pincigher, Giovanna Farruggia, Natalia Calonghi, Gabriele Micheletti

**Affiliations:** 1 Department of Industrial Chemistry ‘Toso Montanari’, Alma Mater Studiorum, Università di Bologna, Viale del Risorgimento 4, 40136 Bologna, Italy; giacomo.drius2@unibo.it (G.D.); carla.boga@unibo.it (C.B.); gabriele.micheletti3@unibo.it (G.M.); 2 Health Sciences and Technologies Interdepartmental Centre for Industrial Research (CIRI SDV), University of Bologna, 40126 Bologna, Italy; 3 Department of Chemistry “Giacomo Ciamician”, University of Bologna, Via Selmi 2, 40126 Bologna, Italy; magda.monari@unibo.it (M.M.); jessica.fiori@unibo.it (J.F.); erika.esposito8@unibo.it (E.E.); 4 IRCCS, Istituto Scienze Neurologiche di Bologna, Via Altura 1/8, 40139 Bologna, Italy; 5 Department of Pharmacy and Biotechnology, University of Bologna, Via San Donato 15, 40127 Bologna, Italy; chiara.zalambani2@unibo.it (C.Z.); pincigherluca@gmail.com (L.P.); giovanna.farruggia@unibo.it (G.F.)

**Keywords:** Ru(II), hydroxy stearic acid, lipophilicity, anticancer, DNA damage, alkyl chain

## Abstract

Metallodrugs represent a combination of multifunctionalities that are present concomitantly and can act differently on diverse biotargets. Their efficacy is often related to the lipophilic features exhibited both by long carbo-chains and the phosphine ligands. Three Ru(II) complexes containing hydroxy stearic acids (HSAs) were successfully synthesized in order to evaluate possible synergistic effects between the known antitumor activity of HSA bio-ligands and the metal center. HSAs were reacted with [Ru(H)_2_CO(PPh_3_)_3_] selectively affording O,O-carboxy bidentate complexes. The organometallic species were fully characterized spectroscopically using ESI-MS, IR, UV-Vis, and NMR techniques. The structure of the compound Ru-12-HSA was also determined using single crystal X-ray diffraction. The biological potency of ruthenium complexes (Ru-7-HSA, Ru-9-HSA, and Ru-12-HSA) was studied on human primary cell lines (HT29, HeLa, and IGROV1). To obtain detailed information about anticancer properties, tests for cytotoxicity, cell proliferation, and DNA damage were performed. The results demonstrate that the new ruthenium complexes, Ru-7-HSA and Ru-9-HSA, possess biological activity. Furthermore, we observed that the Ru-9-HSA complex shows increased antitumor activity on colon cancer cells, HT29.

## 1. Introduction

Despite extensive research and biochemical testing of promising novel treatments, cancer was still responsible for an estimated 9.6 million deaths in 2018 and remains one of the leading causes of death worldwide. In 2023, it is predicted that there will be 609,820 cancer deaths in the United States alone [1,2,3,4]. The issue has been made worse by COVID-19 pandemic diffusion causing remarkable delays in cancer diagnosis and treatments, which may lead to enhanced morbidity and mortality for the next few years [5,6]. Therefore, it is claimed that there is an urgent global need to develop novel potential anticancer agents. In this regard, metal complexes show promise as novel antineoplastic agents against a plethora of different cancer types [7,8,9,10,11,12,13,14,15,16]. Ruthenium metal complexes, due to their tumor cell selectivity and reduced toxicity towards normal cells, constitute a well-established option for anticancer drugs [17,18,19,20,21,22,23]. These phenomena could be explained by the ruthenium ability to mimic the iron center to bind carrier biomolecules as transferrin or albumin [24,25]. In fact, some Ru(III) complexes have been tested in clinical trials, i.e., NAMI-A, KP1019, and NKP-1339 [26,27,28]. However, Ru(II) complexes have demonstrated great antitumor effectiveness mainly due to the facile ligand substitution features, analogous to Pt(II) species [29,30], which are currently in use [31]. The selection of ligands plays a key role in anticancer activity. Mechanisms involving Ru(II) complexes bearing O,O- chelating ligands, indicate interesting chemical and biological properties [32,33,34]. Moreover, ligands with enhanced lipophilicity are likely to afford complexes remarkable efficacy in eukaryotic cell treatment [24,35,36]. On this matter, the addition of the hydrophobic PPh_3_ ligand to Ru(II) centers increases drug uptake and cancer cells’ antiproliferative potential, which may be ascribed to the complex ability to intercalate DNA nucleobase pairs [14,37].

**Figure 1:**
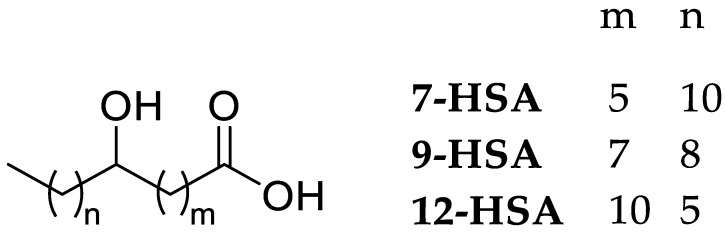
General structures of hydroxy stearic acids (HSAs).

**Scheme 1:**
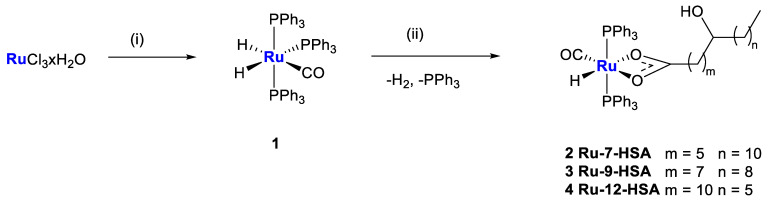
Synthesis of complexes **2**–**4**. (i) PPh_3_, EtOH, KOH, reflux, o/n (ii) HSA, 1,2-DME, reflux, 3–4 h.

In line with the crucial biological activity of hydroxy stearic acids, we recently described the effects of the hydroxyl group position along the 18-carbon chain for several HSA regioisomers [38]. Unexpectedly, the HSAs with the hydroxyl group in the odd position (5-, 7-, 9-, 11-HSA) show inhibitor activity against various human tumor cell lines, while HSAs with the -OH group in the even position (8-, 10-, 12-HSA) display reduced activity. Several hybrid transition metal complexes containing anticancer moieties as ligands have been extensively studied in recent years [14,39,40,41]. Compared to free organic ligands, metallodrugs commonly show a remarkable enhanced anticancer activity [42,43,44,45], mainly ascribed to the advantages that arise due to the increased stability of the molecules and the ligand features. Thus, in combination with the selected metal, the nature of ligands can confer to the molecule the ability to distinctively interact with different cellular targets, thus overcoming tumor affections and minimizing drug resistance [46]. In this context, we selected ligands with well-established antitumor activity (7- and (*R*)-9-HSA) and 12-HSA (see Figure 1) as innocuous candidates to control the non-innocent action exerted by the Ru(II) metal atom in altering the inert properties of the organic molecules. Accordingly, selected ligands were reacted with the ruthenium species *mer*-[Ru(H)_2_CO(PPh_3_)_3_], **1** (Scheme 1) to obtain the expected Ru(II)-HSA derivatives. Hence, the carboxylic moiety can chelate the Ru center, promoting thermodynamically favorite species by concomitant release of molecular hydrogen and PPh_3_, which are easily removable by extraction.

The nature of the metal precursor **1** has been selected with the purpose to obtain lipophilic complexes, which has been recognized to be a key factor related to the enhanced cell uptake [37,47], but also by involving a rather sustainable synthetic procedure, through releasing of H_2_ and PPh_3_. The lipophilic character of the target complexes is given by the structure of the stearic aliphatic chain, but also by the oppositely located PPh_3_ ligands, which results in the encumbered metal-organic scaffold.

The biological potency of ruthenium complexes (Ru-7-HSA, Ru-9-HSA, and Ru-12-HSA; see Scheme 1) was studied on human primary cell lines (HT29, HeLa, and IGROV1). Tests concerning cytotoxicity, cell proliferation, and DNA damage were further performed to provide detailed information about anticancer properties.

## 2. Results and Discussion

### 2.1. Synthesis and Characterization

The first step for the preparation of the Ru(II)-HSA complexes is the synthesis of racemic 7-HSA and (*R*)-9-HSA, while 12-HSA (Figure 1) was purchased as commercially available. 7-HSA and (*R*)-9-HSA were obtained in pure form following the synthetic procedures recently reported [38,48]. The following step involves the reaction of the HSAs with a stoichiometric amount of [Ru(H)_2_CO(PPh_3_)_3_], **1**—obtained according to slightly modified reported processes [49]—and the synthesis selectively leads to the targeted Ru(II)-HSA complexes after purification as a unique species. As shown by Scheme 1, 3 distinct Ru(II) complexes were obtained in moderate yields (35–42%). The final compounds **2–4** were characterized by multinuclear NMR, IR, UV-vis spectroscopy, and mass spectrometry to confirm their nature and purity. In solid state, the complexes are stable to air and light and are soluble in methanol, ethanol, diethyl ether, toluene, chloroform, dichloromethane, and DMSO.

### 2.2. Spectroscopic Studies

The spectroscopic data for the new complexes **2–4** are analogous (Supplementary Materials Figures S1–S21). Hence, the spectroscopic data of **3** are described as an example for this small library of complexes. The IR spectrum of free 9-HSA showed bands at 3408, 3344, and 1698 cm^−1^ corresponding to ν(-OH), ν(COOH), and ν(C=O), respectively. In the IR spectrum of **3** (see Supplementary Materials Figure S11), the band attributed to the hydroxyl moiety appears slightly shifted to 3427 cm^−1^. The disappearance of the band at 3344 cm^−1^, which corresponds to the vibration of the carboxylic moiety (ν(COOH)), provides evidence for the coordination of the carboxylic group to the ruthenium metal center. The sharp band at 1521 cm^−1^ can be assigned to the asymmetric carboxylate vibration, while the symmetric carboxylate vibration at 1454 cm^−1^ is partially covered by the P-C band at 1433 cm^−1^. The reduced difference of 67 cm^−1^ between the asymmetric and symmetric stretches of the carboxylate group can be assigned to the chelate binding mode, according to the literature [50,51]. The absorption at 2084 cm^−1^ has instead been assigned to the H-Ru vibration. The existence of a peak around 1940 cm^–1^ in the precursor indicates the presence of a terminally coordinated carbonyl ligand. Upon coordination of 9-HSA ligand, this strong and sharp band is shifted to 1913 cm^−1^. The absorptions at 2925 and 2853 cm^−1^ can be respectively attributed to methyl (-CH_3_) and methylene (-CH_2_-) functional groups of the 9-HSA ligand, while the weak bands at 3057 cm^−1^ are due to the aromatic C-H stretching of PPh_3_ [52]. The absorption peak at 270 nm observed in the electronic spectrum of complex **3** is a clue point for the coordination to the metal (see Supplementary Materials Figure S14). The absorption is commonly attributed to the n → π* transition, which is instead a forbidden transition in the case of the free ligand [53].

The ^1^H NMR of the precursor, free HSA ligand, and Ru(II) complexes were recorded to support the presence of the coordinated carboxylic unit to the new Ru(II) complexes. The spectrum of complex **3** (see Supplementary Materials Figure S8) in the range 10–13 ppm shows no evidence of signals due to carboxylic protons. The free organic 9-HSA ^1^H spectrum presents a multiplet at 3.66–3.54 ppm and aliphatic proton signals can be found between 2.37 and 0.89 ppm. Upon coordination to the metal, a broad signal can be observed at 3.55 ppm and other signals are found in the δ = 1.45–0.55 range. Spectrum integration confirms the presence of 34 protons of the HSA coordinated ligand. Two ^1^H NMR signals are observed respectively at −6.50 and −8.30 ppm in the precursor spectrum assigned to the high shifted hydride ligands, whereas in the spectrum of complex **3** only one signal can be observed at −16.39 ppm, confirming the replacement of Ru-H and a PPh_3_ by the carboxy group. The hydride signal is a triplet (^2^*J*_HP_ = 20.6 Hz) due to the coupling with the 2 equivalent P atoms in a reciprocally *trans* position. All Ru-HSA complexes show multiplets in the range 7.69–7.10 ppm due to the presence of aromatic protons of triphenylphosphines. The ^13^C NMR spectrum of complex **3** (see Supplementary Materials Figure S10) displays a downfield triplet (^2^*J*_CP_ = 10.5 Hz) at 205.61 ppm that can be assigned to the terminal Ru carbonyl moiety. The next downfield singlet at 186.20 ppm is assigned to the carboxylic carbon atom. The aryl carbons are found to resonate in the range 134.48−128.16 ppm. A singlet at 72.09 ppm is assigned to the asymmetric CH-OH carbon atom. The signals relating to the aliphatic HSA ligand chain are observed in the range 37.62−14.21 ppm. The ^31^P NMR spectra were recorded to confirm the purity of derivatives and the geometry of the two PPh_3_ in the complexes. The ^31^P NMR spectrum of complex **3** (see Supplementary Material Figure S9) shows a doublet (^2^*J*_HP_ = 18.8 Hz) at 44.53 ppm, confirming the magnetically equivalence of the two phosphorous atoms and the *trans* position to each other around the metal center. ESI-MS spectra were recorded in positive ion mode and showed [M − H]^+^ at 953, [M + H]^+^ at 955, [M + Na]^+^ at 977, and [M + K]^+^ at 994 *m/z* (see Supplementary Materials Figures S12 and S13). The sample for mass analysis was dissolved in acetonitrile; for this reason, it is possible to observe in the ESI mass spectra very intense peaks with the isotopic distribution of ruthenium at 696 [M − 9-HSA + CH_3_CN] and 737 *m/z* [M − 9-HSA + 2CH_3_CN]^+^.

### 2.3. Crystal Structure Description of ***4***

**Figure 2:**
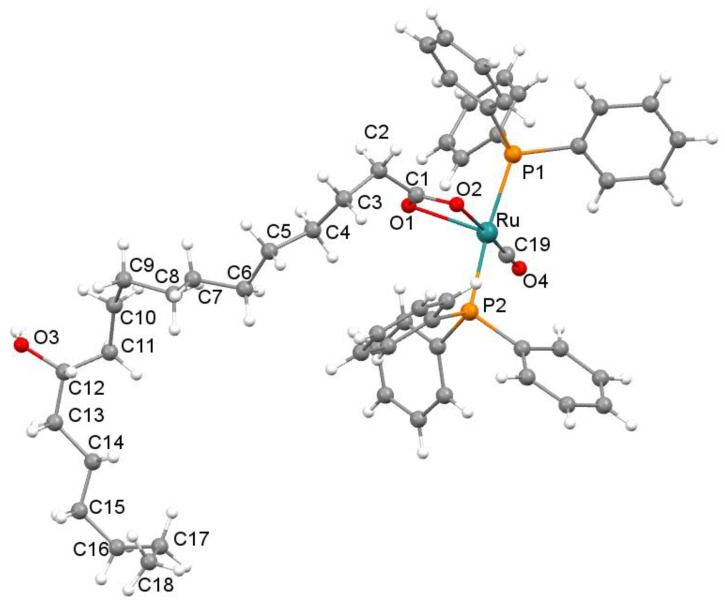
Molecular structure of **4**.

X-ray structure determination of free 9-, 10-, and 12-hydroxy stearic acids has been reported [54,55,56,57]. The X-ray molecular structure of **4** is shown in Figure 2 (crystal data are reported in Table S1).

**Figure 3:**
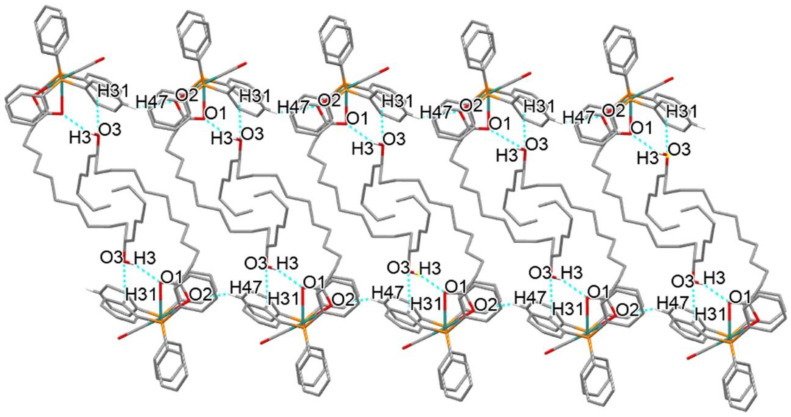
Arbitrary view of the crystal packing of **4**. For the sake of clarity only the hydrogens engaged in H bonding (light blue dotted lines) are reported.

The coordination geometry at the Ru atom is distorted octahedral with the PPh_3_ ligands in *trans* configuration, one CO and two O atoms of the carboxylate moiety of the 12-HSA anion chelating the metal center. The hydride completing the Ru coordination sphere could not be located but its presence can be deduced from the wide C(19)-Ru-O(2) angle [174.0(5)°] and from the ^1^H NMR spectrum signal (see Supplementary Material Figure S15). The two Ru-O distances [Ru-O(2) and Ru-O(1) 2.177(9) and 2.307(9) Å, respectively] are significantly different, presumably because of the *trans* influence exerted by the hydride. The long aliphatic chain of the 12-hydroxy-stearate is bent due to the formation of two intermolecular O3-H3…O1 hydrogen bonds between the hydroxy hydrogen and one carboxylate oxygen of an adjacent molecule (Figure 3) with formation of dimeric units. In addition, non-classical C-H…O interactions involving the hydroxy oxygen and one aromatic hydrogen [C31-H31…O3] of a neighbor molecule or one aromatic hydrogen and the other carboxylate oxygen O2 [C47-H47…O2] form a chain of dimeric units (see Supplementary Material Table S2). The supramolecular network is completed by π-π interactions between phenyl rings of different chains of dimers, as shown in Figure S24.

### 2.4. Stability Studies in Solution

Complexes **3** and **4** were used as a reference to determine the stability in solution of this class of compounds. To provide qualitative insight into the stability of complexes at physiological pH in solution phase, their UV–Vis spectra were recorded in phosphate buffer solution (PBS-5% DMSO) over a period of 48 h, according to the literature [58,59]. The spectra of the complexes show no wavelength shifts, indicating that their structural stability was maintained throughout the experiment (see Supplementary Materials Figures S22 and S23).

### 2.5. Lipophilicity Evaluation of ***2***–***4***

The partition coefficient (P) describes the propensity of a neutral compound to dissolve in an immiscible biphasic system composed of an organic solvent and water. Lipophilicity of a potential drug can be evaluated by the n-octanol-water partition coefficient (log P_o_/_w_). Generally, log P_o_/_w_ values included between 0 and 3 constitute an ideal range for passive drug absorption, while values lower than 0 are given by highly hydrophilic compounds with scarce cell permeability [47,60]. Using the shake-flask technique, the log P_o/w_ values of 0.95 ± 0.19, 0.71 ± 0.08, and 0.85 ± 0.06 were respectively obtained for **2**, **3**, and **4**, confirming the lipophilicity of the complexes.

### 2.6. Antiproliferative Activity

Primary screening of antiproliferative activity of the ruthenium complexes **1**, **2**, **3**, and **4** was performed by the commonly used MTT assay on three cancer cell lines of various origins: ovarian (IGROV1), cervical (HeLa), and colon (HT29). In addition, human dermal fibroblasts (HDFa) were used to assess the toxicity of the complexes on a normal cell line. The results are summarized in Table 1. The IC_50_ values obtained for the complexes **2**–**4** are compared with those obtained for [Ru(H)_2_CO(PPh_3_)_3_] **1** and cisplatin (**CDDP**) used under the same experimental conditions.

**Figure 4:**
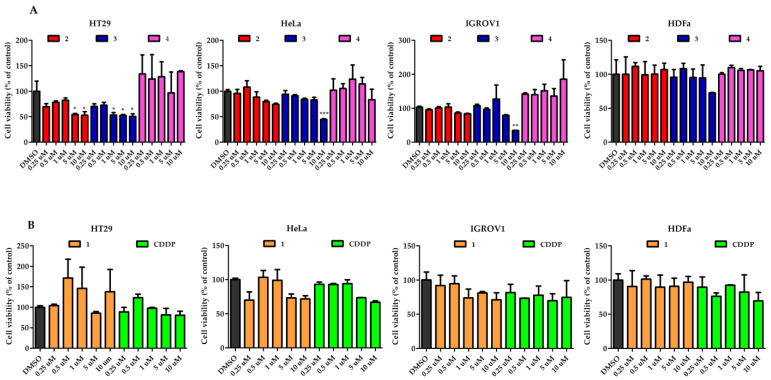
The percentage cell viability of human malignant and non-malignant cells analyzed with an MTT viability assay. (**A**) The cells were treated for 48 h with a concentration range of 0.25–10 μM with compounds **2**, **3**, and **4** dissolved in DMSO (vehicle control). (**B**) Compound **1** or **CDDP** (positive control) at the same concentrations was also included. Error bars are standard deviations. Significant differences are indicated as * *p* < 0.05, ** *p* < 0.01, and *** *p* < 0.001.

Dose–response graphs were constructed to determine the IC_50_ concentrations of various treatments including **CDDP** (Figure 4A,B). The results presented in Table 1 and Figure 4 indicate that complexes **2** and **3** were cytotoxic in malignant cell lines, acting in different ways. Indeed, in HT29 complex **2** caused a decrease in cell viability at the concentration of 5 and 10 µM, while **3** was more potent, inducing a cytotoxic effect already at 1 µM. In HeLa and IGROV1, only complex **3** was active at the concentration of 10 µM.

Complex **4** showed no effect on cell viability in all lines tested. All Ru-HSAs complexes were less toxic to non-malignant HDFa cells. Complex **1** and **CDDP** had no significant effect on both non-malignant and malignant cells (Figure 4B).

Following cell viability testing, the IC_50_ concentrations were determined for both the non-malignant and malignant cell models (Table 1).

Some authors [47,61] have synthesized and characterized five lipophilic Ru(III) complexes which differ in their lipid tail, demonstrating how some of the lipophilic Ru(III) complexes show promising antiproliferative properties in vitro on a selected panel of tumor cells and no significant toxicity to healthy cells. In particular, the PalmiPyRu complexes and StePyRu proved to be the most effective in reducing cell growth and proliferation of MCF-7 breast cancer cells. The authors believe these results could be explained by their long and saturated lipid chain, which can probably ensure a more efficient structuring of their aggregates in aqueous solutions, favoring the protection of the ruthenium core [47,61]. However, in our case, the antitumor activity observed for **2** and **3** compared with **1** cannot be attributed to their lipophilicity alone. Interestingly, the position of the hydroxyl moiety in the long chain drastically influences the in vitro potency. In general, Ru-7-HSA and Ru-9-HSA showed statistically significant inhibitory potency at concentrations lower than or equal to 10 μM, whereas when the hydroxyl group is at position 12 of the stearic chain (Ru-12-HSA), no activity was observed. Furthermore, the observation that complex **3** is active in all tested malignant lines, while complex **2** acts only in HT29, suggests that in vitro potency is also influenced by the characteristics of individual cell lines.

### 2.7. Cell Cycle Analysis by Quantification of DNA Content

**Figure 5:**
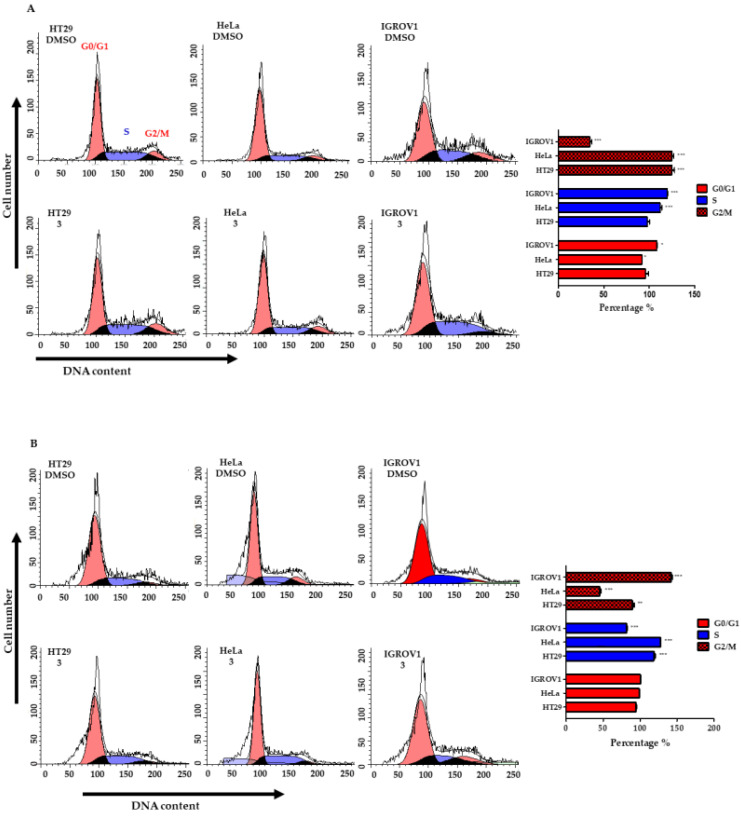
Flow cytometric analysis of control and treated cells with 10 µM of **3**. (**A**) (**Left**) HT29, HeLa, and IGROV1 were treated with 10 μM of complex **3** for 24 h and then were analyzed using a flow cytometer. (**Right**) The percentage of cells in the different cell cycle phases was calculated at 24 h. (**B**) (**Left**) HT29, HeLa, and IGROV1 were treated with 10 μM of complex **3** for 48 h and then were analyzed using a flow cytometer. (**Right**) The percentage of cells in the different cell cycle phases was calculated at 48 h. Error bars are standard deviations. Significant differences are indicated as * *p* < 0.05, ** *p* < 0.01, and *** *p* < 0.001.

To determine whether the antiproliferative activity of **3** was also responsible for growth arrest or retardation in a particular phase of the cell cycle, cells were stained with PI after 24 and 48 h of treatment with the complex at the concentration of 10 μM followed by flow cytometric analysis. According to flow cytometry histogram statistics, treatment of cells with **3** induces a population shift during the cell cycle relative to the control, as clearly reported in Figure 5A. Figure 5B shows the graphs relative to the percentage changes in the different cell cycle phases. In HT29, treatment for 24 h with **3** increased the percentage of cells in G2/M phase by 25.0% ± 5.0, while after 48 h it induced an accumulation in S phase by 18.7% ± 2.7 followed by a reduction in G2/M by 10.5% ± 4.2. In HeLa, 24 h treatment with **3** decreased the percentage of cells in G0/G1 by 8.1% ± 0.7, while it increased in both S-phase and G2/M by 12.0% ± 3.3, and 24.9% ± 3.7, respectively. After 48 h of treatment with **3**, the percentage of HeLa in the S phase was 27.4% ± 0.9, while the G2/M decreased by 45.3% ± 2.7. In IGROV1, treatment for 24 h with **3** increased the percentage of cells in S phase and decreased that in G2/M by 19.9% ± 0.9 and 34.8% ± 3.5, respectively. At 48 h the antiproliferative effect continued with a significant accumulation in G2/M phase equal to 41.8% ± 3.1. Taken together, these results indicated that the Ru-9-HSA complex, at the concentration of 10 µM, causes a cytotoxic effect in all neoplastic lines characterized by an accumulation in the S and G2/M cell cycle phases that persists over time.

### 2.8. Complex 3 Induced DNA Damage in HT29 Cells

Prior to this study, we reported that 9-HSA upregulates p21WAF1 in HT29 tumor cells and induces an arrest in the G0/G1 phase of the cell cycle by targeting histone deacetylase 1 [62,63]. Other authors have demonstrated that 9-HSA induces the arrest of the cell cycle but does not promote apoptosis [64]. These findings suggest that the biological effect induced by **3** cannot be attributed to 9-HSA. Indeed, the Ru-9-HSA at 24 h of treatment causes an accumulation of cells in the G2/M phase, while after 48 h the damage continues with an accumulation of cells in the S phase. Genotoxic agents, including ionizing radiation (IR), induce a variety of DNA injuries, with DNA double-strand breaks (DSBs) being the most deleterious type of damage, if not properly repaired. The first and most prominent protein for which foci formation at the site of a DSB was described is the histone variant H2AX, which is phosphorylated at its C-terminal Ser-139 residue by the DNA damage-activated kinases to form γH2AX [65,66].

Using γH2AX levels as a measure of DNA damage, particularly that of DNA DSBs, previous studies have shown that DNA damage peaks in the S, G2/M phases of the cell cycle with a variety of genotoxic treatments [67,68,69].

**Figure 6:**
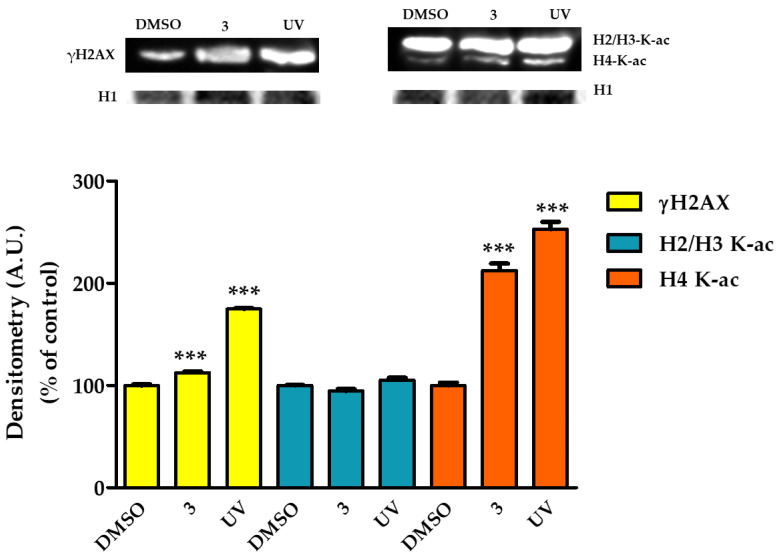
(**Above left**) Representative Western blot image of γH2AX in HT29 cells treated with **3** (10 µM) for 6 h or exposed to UV for 3 min. (**Above right**) Representative image of acetylation status of histones H2/H3 and H4. (**Below**) Relative quantification of γH2AX and histone acetylation. Arbitrary densitometry units (A.U.) were normalized by H1 histone. All data represent mean ± SD (*n* = 3). *** *p* < 0.001, compared with controls.

This has established γH2AX as a standard, direct, and faithful marker of DNA damage inside a cell. We studied the effects of **3** on DNA damage by evaluating the phosphorylation status of H2AX in a Western blot. UV-irradiated HT29 were used as a positive control. Cells were treated with vehicle or 10 μM complex **3** for 6 h, and extracted histones were analyzed for the presence of γH2AX. As shown in Figure 6, treatment with complex **3** and UV significantly increased γH2AX levels compared with no treatment. Regarding acetylation, both treatments significantly increased histone H4 acetylation, indicating that the chromatin of cells undergoes an active change after UV and **3** treatment that could trigger the activation of DNA damage repair mechanisms.

## 3. Materials and Methods

### 3.1. Chemical Synthesis

#### 3.1.1. General

All reactions were routinely carried out under argon atmosphere, using standard Schlenk techniques. Solvents were HPLC grade and degassed before use. Glassware was oven dried before use. Infrared spectra (4000–400 cm^−1^) were recorded at 298 K on a PerkinElmer Spectrum 2000 FT-IR (Fourier transform infrared) spectrophotometer (Waltham, MA, USA), and ESI MS (electrospray ionization mass spectrometry) spectra were recorded on a Waters Micromass ZQ 4000 (Milford, MA, USA), with samples dissolved in CH_3_OH or CH_3_CN. All deuterated solvents were degassed before use. NMR measurements were taken on Varian Inova 300 (Varian, Palo Alto, CA, USA), a Mercury Plus 400 (Oxford Instruments, Abingdon-on-Thames, UK), and an Inova 600 (Varian, Palo Alto, CA, USA) instruments. Frequencies are reported in Hz and the chemical shifts were referenced to the solvent (CDCl_3_ δ = 7.27 and 77.0 ppm for ^1^H and ^13^C NMR, respectively). NMR spectra were recorded at 298 K. The chemical shifts are expressed in parts per million (ppm). Absorption spectra were recorded using an Agilent Cary 100 UV–vis spectrometer (Santa Clara, CA, USA). All the chemicals were of reagent grade and were used as received from commercial suppliers. Commercially available [RuCl_3_·xH_2_O] was purchased from Strem (Bischheim, France). Compound [Ru(H)_2_CO(PPh_3_)_3_] **1** was prepared by published methods [49]. Commercially available 12-HSA was purchased from Merk (Darmstad, Germany). 7-, 9-HSA were prepared by published methods [38,48].

#### 3.1.2. Synthesis of [RuH(CO)12-HSA(PPh_3_)_2_]

The ligand 12-HSA (49 mg, 0.163 mmol) and [Ru(H)_2_CO(PPh_3_)_3_] **1** (150 mg, 0.163 mmol) were dissolved in 1,2-dimetoxy ethane (10 mL) and refluxed for 4 h. The solution was cooled to room temperature and 1,2-dimetoxy ethane was evaporated under vacuum. The solid was dissolved in a minimum volume of diethyl ether, and after the addition of 15 mL of petroleum ether, a white solid precipitated. This was filtered and washed 3 times with 10 mL of petroleum ether and dried.

[RuH(CO)12-HSA(PPh_3_)_2_] (**4**, Ru-12-HSA): 42% yield (66 mg, 0.069 mmol). FT-IR (cm^−1^) in KBr: 3414 *ν* (-OH), 3056 *ν*(aromatic C-H stretch), 2929 *ν*(methylene C-H asym. stretch), 2853 *ν*(methylene C-H sym. stretch), 2083 *ν*(Ru-H), 1913 *ν*(C≡O), 1521 *ν*(asym. COO), 1457 *ν*(sym. COO), 1434, 1094, 692 *ν*(PPh_3_). UV-vis (DMSO), λ_max_, nm: 270. ^1^H NMR (300 MHz, CDCl_3_) δ (ppm) 7.71–7.46 (12 H, m, PPh_3_), 7.44–7.29 (18 H, m, PPh_3_), 3.58 (1 H, bs, CH-OH), 1.42 (5 H, bs), 1.33–1.12 (15 H, m), 1.09–0.97 (2 H, m), 0.96–0.81 (7 H, m), 0.77–0.62 (2 H, m), 0.57 (2 H, m), −16.39 (1 H, t, *J* = 20.7 Hz, Ru-H). ^13^C NMR (101 MHz, CDCl_3_) δ (ppm) 205.61 (t, ^2^*J_CP_* = 10.0 Hz, C≡O), 186.24 (COO-Ru), 134.89–128.20 (30 C, aromatic PPh_3_), 72.17 (CH-OH), 42.98, 37.66, 36.89, 36.17, 31.99, 29.88–29.14, 25.83, 25.77, 24.28, 23.25, 22.77, 14.24. ^31^P NMR (121 MHz, CDCl_3_): δ (ppm) 44.56 (bs, 2P). ESI-MS (*m/z*): 955 [M + H]^+^.

#### 3.1.3. Synthesis of [RuH(CO)9-HSA(PPh_3_)_2_]

The ligand 9-(*R*)-HSA (36 mg, 0.120 mmol) and [Ru(H)_2_CO(PPh_3_)_3_] **1** (110 mg, 0.120 mmol) were dissolved in 1,2-dimetoxy ethane (8 mL) and refluxed for 3 h. The solution was cooled to room temperature and 1,2-dimetoxy ethane was evaporated under vacuum. The solid was dissolved in 5 mL of diethyl ether and filtered on celite. Diethyl ether was evaporated, and the purple solid was partially dissolved in 2 mL of CH_3_CN and cooled to −20 °C. After 20 min this was filtered and washed with 2 mL of cold CH_3_CN and dried.

[RuH(CO)9-HSA(PPh_3_)_2_] (**3**, Ru-9-HSA): 35%s yield (40 mg, 0.042 mmol). FT-IR (cm^−1^) in KBr: 3427 *ν* (-OH), 3057 *ν*(aromatic C-H stretch), 2925 *ν*(methylene C-H asym. stretch), 2853 *ν*(methylene C-H sym. stretch), 2084 *ν*(Ru-H), 1913 *ν*(C≡O), 1521 *ν*(asym. COO), 1454 *ν*(sym. COO), 1433, 1095, 692 *ν*(PPh_3_). UV-vis (DMSO), λ_max_, nm: 270. ^1^H NMR (400 MHz, CDCl_3_) δ (ppm) 7.68–7.46 (12 H, m, PPh_3_), 7.47–7.28 (18 H, m, PPh_3_), 3.55 (1 H, bs, CH-OH), 1.48–1.34 (5 H, m), 1.33–1.20 (15 H, m), 1.13–1.00 (2 H, m), 0.99–0.84 (7 H, m), 0.75–0.63 (2 H, m), 0.63–0.51 (2 H, m), −16.39 (1 H, t, *J* = 20.6 Hz, Ru-H). ^13^C NMR (151 MHz, CDCl_3_) δ (ppm) 205.61 (t, ^2^*J_CP_* = 10.5 Hz, C≡O), 186.20 (COO-Ru), 134.48–128.16 (30 C, aromatic PPh_3_), 72.09 (CH-OH), 37.62, 36.86, 32.06, 31.96, 29.85–29.10 (6 signals), 25.80, 25.74, 24.25, 23.65, 22.73, 14.21. ^31^P NMR (162 MHz, CDCl_3_): δ (ppm) 44.53 (d, ^2^*J*_PH_ = 18.8 Hz, 2P). ESI-MS (*m/z*): 953 [M − H]^+^, 955 [M + H]^+^, 977 [M + Na]^+^, 994 [M + K]^+^.

#### 3.1.4. Synthesis of [RuH(CO)7-HSA(PPh_3_)_2_]

The ligand 7-HSA (30 mg, 0.100 mmol) and [Ru(H)_2_CO(PPh_3_)_3_] **1** (92 mg, 0.100 mmol) were dissolved in 1,2-dimetoxy ethane (8 mL) and refluxed for 3 h. The solution was cooled to room temperature and 1,2-dimetoxy ethane was evaporated under vacuum. The solid was dissolved in minimum volume of diethyl ether, and after the addiction of 15 mL of *n*-heptane, a light-brown solid precipitated. This was filtered and washed 3 times with 5 mL of *n*-heptane and dried.

[RuH(CO)7-HSA(PPh_3_)_2_] (**2**, Ru-7-HSA): 42% yield (40 mg, 0.042 mmol). FT-IR (cm^−1^) in KBr: 3383 *ν* (-OH), 3058 *ν*(aromatic C-H stretch), 2925 *ν*(methylene C-H asym. stretch), 2852 *ν*(methylene C-H sym. stretch), 1921 *ν*(C≡O), 1521 *ν*(asym. COO), 1481 *ν*(sym. COO), 1434, 1094, 692 *ν*(PPh_3_). UV-vis (DMSO), λ_max_, nm: 261. ^1^H NMR (400 MHz, CDCl_3_) δ (ppm) 7.69–7.10 (30 H, m, PPh_3_), 3.44 (1 H, s, -OH), 3.38 (1 H, bs, CH-OH), 1.36–1.18 (24 H, m), 0.94–0.84 (5 H, m), 0.79–0.65 (2 H, m), 0.65–0.50 (2 H, m), −16.41 (1 H, t, *J* = 20.6 Hz, Ru-H). ^13^C NMR (101 MHz, CDCl_3_) δ (ppm) 205.48 (t, ^2^*J_CP_* = 10.0 Hz, C≡O), 188.87 (COO-Ru), 136.88–127.06 (30 C, aromatic PPh_3_), 71.98 (CH-OH), 37.67, 37.25, 36.74, 32.05, 29.88–28.78, 25.80, 25.37, 25.30, 24.25, 23.25, 22.82, 14.26. ^31^P NMR (162 MHz, CDCl_3_): δ (ppm) 44.49 (d, ^2^*J_PH_* = 18.8 Hz, 2P). ESI-MS (*m/z*): 953 [M − H]^+^, 977 [M + Na]^+^, 994 [M + K]^+^.

### 3.2. Lipophilicity Evaluation

The log P_o/w_ values were determined by the shake-flask method, according to the reported procedure [47]. *N*-octanol solutions (V = 3.0 mL) of complexes **2**, **3**, **4** were prepared at a known concentration in the range $5.0\;\cdot 10^{4}-6.5\;\cdot 10^{4}$ M. Thus, an equal volume (3.0 mL) of PBS was added to n-octanol and each solution was shaken at room temperature for 2 h, and was left to equilibrate for 30 min. The phases were separated, and the organic solution was analyzed by UV–Vis spectroscopy after proper dilution. The log P_o/w_ values were calculated according to the Lambert–Beer Law. Each experiment was repeated three times.

### 3.3. X-ray Crystallography

The X-ray intensity data were collected on a Bruker Apex II CCD diffractometer (Karlsruhe, Germany). The SMART software was used for gathering frames of data, indexing reflections and determination of lattice parameters. The collected frames were then processed for integration by the SAINT program, and an empirical absorption correction was applied using SADABS [70]. The structure was solved by direct methods (SHELXT) [71] and subsequent Fourier syntheses and refined by full-matrix least-squares on F2 (SHELXTL) [72] using anisotropic thermal parameters for all non-hydrogen atoms.

CCDC 2254341 contains the supplementary crystallographic data for this paper.

These data can be obtained free of charge via www.ccdc.cam.ac.uk/conts/retrieving.html (accessed on 6 April 2023), or by contacting The Cambridge Crystallographic Data Centre, 12 Union Road, Cambridge CB2 1EZ, UK; fax: +44 1223 33603.

### 3.4. Biology

#### 3.4.1. Malignant and Non-Malignant Cells Culture

HT29 human colorectal adenocarcinoma cells, HeLa human cervix adenocarcinoma cells, and HDFa human adult dermal fibroblasts as control cell lines were purchased from American Type Culture Collection (ATCC, Manassas, VA, USA). The IGROV1 human ovarian cancer cell line was kindly provided by Prof. Colnaghi (Istituto Nazionale Tumori, IRCCS, Milan, Italy). Cells were cultured in RPMI 1640 medium (Labtek Eurobio, Milan, Italy), and supplemented with 10% FCS (Euroclone, Milan, Italy) and 2 mM L-glutamine (Sigma-Aldrich, Milan, Italy), at 37 °C, and a 5% CO_2_ atmosphere. The complexes were dissolved in DMSO in a 30–40 mM stock solution. In cell treatments, the final DMSO concentration never exceeded 0.1%.

#### 3.4.2. MTT Assay

Cells were seeded at 1.5 × 10^4^ cells/well in a 96-well culture plastic plate (Sarsted, Milan, Italy), and after 24 h of growth were exposed to increasing concentrations of each distinct compound (from 0.25 μM to 10 μM) solubilized in RPMI 1640 medium. Controls were included and cells were either treated with DMSO (vehicle control) or a positive control, cisplatin (Molekula, Dorset, UK). For the **CDDP**, the cells were treated at the same concentration range as the complexes. Treatments were left for 48 h to ensure efficient cellular uptake.

MTT assay was performed according to the literature [73]. The absorbance at 570 nm was measured using a multi-well plate reader (Tecan, Männemorf, Switzerland), and data were analyzed by Prism GraphPad software. Percent cell viability was determined with respect to the control. All concentrations were tested in triplicate, and the experiment was repeated three times.

#### 3.4.3. Cell Cycle Analysis

HT29, HeLa, and IGROV1 were plated at a density of 2 × 10^4^ cells/cm^2^ in a Petri dish and after 24 h treated with 10 μM Ru-9-HSA for 24 h or 48 h. The samples were prepared according to Calonghi [48]. In brief, untreated and treated cells were detached and washed in PBS, and the pellet was finally re-suspended in 0.01% Nonidet P-40 (Sigma-Aldrich, Milan, Italy), 10 μg/mL RNase (Sigma-Aldrich, Milan, Italy), 0.1% sodium citrate (Sigma-Aldrich, Milan, Italy), and 50 μg/mL propidium iodide (PI) (Sigma-Aldrich, Milan, Italy), for 30 min at room temperature in the dark. Propidium iodide (PI) fluorescence was acquired on a linear scale and analyzed by Modfit software version 5.2 (San Jose, CA, USA). Flow cytometric assays were performed on a Brite HS flow cytometer (Bio-Rad, Watford, UK) equipped with a Xe/Hg lamp.

#### 3.4.4. Histone Post-Translational Modification

HT29 cells were seeded in a dish and after 72 h treated for 6 h with compound Ru-9-HSA at a final concentration of 10 µM. As a positive control we treated cells with UV radiation, which induces global DNA damage. Cells were cultured as above and after 72 h they were irradiated with a UV lamp 7.5 Watt for 3 min. Cells were harvested and washed with 10 mM sodium butyrate in PBS, and nuclei were isolated according to Amellem and Micheletti [74,75]. The nuclear pellet was suspended in 0.1 mL ice-cold H_2_O using a Vortex mixer, and concentrated H_2_SO_4_ was added to the suspension to give a final concentration of 0.4 N. After incubation at 4 °C for 1 h, the suspension was centrifuged for 5 min at 14,000× *g*, and the supernatant was taken and mixed with 1 mL of acetone. After overnight incubation, the coagulate material was collected by microcentrifugation and air dried. This acid soluble histone fraction was dissolved in 20 μL of H_2_O. Proteins were quantified using a protein assay kit (Bio-Rad, Hercules, CA, USA). Histones were detected by resolving samples on a 10% gel in MES buffer at 200 V for 35 min. Western blotting was performed in transfer buffer at 100 V for 1 h. The nitrocellulose membrane was incubated with anti-acetylated lysines (Millipore, Billerica, MA, USA) or anti γH2AX (Santa Cruz, CA, USA) primary antibodies for 1 h. After five washes with PBS-TWEEN 20 0.1%, the membrane was incubated as before with secondary HRP-conjugated antibody (GE Healthcare, Milan, Italy). After washes with PBS-TWEEN 20 0.1%, antibody binding was detected using an Amersham ECL Plus Western Blotting Detection System (GE Healthcare, Milan, Italy).

#### 3.4.5. Statistical Analysis for Biological Studies

For the dose–response graphs, the average percentages (*n* = 3) were calculated with respect to the vehicle (100%). The effects of the treatments were deemed significant, with respect to the vehicle, with *p*-values of either * *p* < 0.05, ** *p* < 0.01 or highly significant at *** *p* < 0.001, as determined by the Student *t*-test.

## 4. Conclusions

The identification of metal drugs as alternatives to platinum compounds currently administered in the clinical treatment of various types of tumors is an ultimate goal of research. Ruthenium complexes have aroused great interest in this regard due to their versatile anticancer activity and low toxicity of the metallic element.

By seeking effective lipophilic organometallic anticancer candidates, three hydroxy stearic acids, two of which have known antiproliferative properties, were selected and coordinated to the Ru(II) center. By reacting HSAs with the precursor *mer*-[Ru(H)_2_CO(PPh_3_)_3_], three novel Ru(II) complexes, where the ligand is coordinated to the metal center in a bidentate fashion through the carboxylate oxygen atoms, were synthesized in satisfactory yields and fully characterized. Further, the X-ray crystal structure of complex **4** evidences two distinct [OH…OC(O)…H(Ph)PPh_2_] intermolecular H-binding interactions. Crystal packing reveals intermolecular H-interactions by the Ru-carboxyl moiety and the lipophilic phenyl phosphine ligands. The biological potency of ruthenium complexes (Ru-7-HSA, Ru-9-HSA, and Ru-12-HSA) was studied on human primary cell lines (HT29, HeLa, and IGROV1). The effects of 9-HSA, particularly of the (*R*)-9-HSA enantiomer, have been well studied in the HT29 cell line. In these cancer cells, (*R*)-9-HSA upregulates p21WAF1 [62], inhibits cell growth by targeting histone deacetylase 1 [63], and interferes with EGF signaling [76]. A quantity of 50 μM (*R*)-9-HSA leads to dissociation of the HDAC1/cyclinD1 complex, resulting in an arrest in the G0/G1 phase of the cell cycle [76]. These results suggest that the biological effect induced by **3** cannot be attributed to 9-HSA. Indeed, 10 μM Ru-9-HSA causes DNA damage, resulting in accumulation of cells in S and G2/M. The experimental data presented in this work are preliminary and do not allow us to state whether there is a synergistic mechanism of action between 9-HSA and ruthenium in inducing the biological effects observed in HT29. However, these results suggest that 9-HSA promotes cellular internalization of the complex. In addition, the finding that Ru-7-HSA and Ru-9-HSA have significant inhibitory potency, while Ru-12-HSA is inactive, suggests that the position of the hydroxyl group along the aliphatic chain may drastically affect the internalization of the complexes and, consequently, the in vitro potency.

Overall, our results demonstrate that the newly synthesized ruthenium complexes (Ru-7-HSA and Ru-9-HSA) possess potential biological activity, especially in the case of Ru-9-HSA.

## Figures and Tables

**Table 1:** Evaluation of the antiproliferative activity of **2**, **3**, **4**, **1**, and **CDDP** on human cancer (HT29, HeLa, and IGROV1) and healthy cell line (HDFa) after 48 h incubation. IC_50_ (μM) values are reported as mean ± SD values of at least three independent experiments.

Complex	HT29	HeLa	IGROV1	HDFa
**2**	2.43 ± 0.01	>10	>10	>10
**3**	0.17 ± 0.02	10.43 ± 0.05	8.55 ± 0.01	>10
**4**	>10	>10	>10	>10
**1**	>10	>10	>10	>10
**CDDP**	>10	>10	>10	>10

## Data Availability

Not applicable.

## Supplementary Materials

The following supporting information can be downloaded at: https://www.mdpi.com/article/10.3390/molecules28104051/s1. Figures S1–S7: Characterization of **2**; Figures S8–S14: Characterization of **3**; Figures S15–S21: Characterization of **4**; Figures S22 and S23: Stability studies of complexes **3** and **4** in solution; Figure S24 and Tables S1 and S2: X-ray Crystallography.

## Sample Availability

Samples of some compounds are available from the authors.
