# Supplementary material for: Synthesis and Antiproliferative Insights of Lipophilic Ru(II)-Hydroxy Stearic Acid Hybrid Species
Source: Molecules. 2023 May 12;28(10):4051. doi: 10.3390/molecules28104051 (PMC10222286; doi:10.3390/molecules28104051)
Supplement: Supplementary file 1 [file molecules-28-04051-s001.zip › molecules-2363220-supplementary.pdf]

Synthesis and Antiproliferative Insights of Lipophilic Ru(II)-Hydroxy Stearic Acids Hybrid Species

Giacomo Drius [1], Silvia Bordoni [1,2,*], Carla Boga [1], Magda Monari [3], Jessica Fiori [3], Erika Esposito [3,4], Chiara Zalambani [5], Luca Pincigher [5], Giovanna Farruggia [5], Natalia Calonghi [5,*] and Gabriele Micheletti [1]

[1] Department of Industrial Chemistry 'Toso Montanari', Alma Mater Studiorum, Università di Bologna, Viale del Risorgimento 4, 40136 Bologna, Italy; giacomo.drius2@unibo.it (G.D.); silvia.bordoni@unibo.it (S.B.); carla.boga@unibo.it (C.B.); gabriele.micheletti3@unibo.it (G.M.)
[2] Health Sciences and Technologies Interdepartmental Center for Industrial Research (CIRI SDV), University of Bologna, 40126 Bologna, Italy
[3] Department of Chemistry "Giacomo Ciamician", University of Bologna, Via Selmi 2, 40126 Bologna, Italy; magda.monari@unibo.it (M.M.); jessica.fiori@unibo.it (J.F.); erika.esposito8@unibo.it (E.E.)
[4] IRCCS, Istituto Scienze Neurologiche di Bologna, Via Altura 1/8, 40139 Bologna, Italy
[5] Department of Pharmacy and Biotechnology, University of Bologna, Via San Donato 15, 40127 Bologna, Italy; chiara.zalambani2@unibo.it (C.Z.); farruggia.giovanna@unibo.it (F.G.) natalia.calonghi@unibo.it (N.C.); pincigherluca@gmail.com (L.P.)
* Correspondence: natalia.calonghi@unibo.it, silvia.bordoni@unibo.it

Contents

Characterization of **2 (Ru-7-HSA)** 2
- NMR spectra of **2** 2
- IR spectrum of **2** 4
- Mass Spectra of **2** 4
- UV-vis spectrum of **2** 6

Characterization of **3 (Ru-9-HSA)** 7
- NMR spectra of **3** 7
- IR spectrum of **3** 9
- Mass Spectra of **3** 11
- UV-vis spectrum of **3** 12

Characterization of **4 (Ru-12-HSA)** 13
- NMR spectra of **4** 13
- IR Spectrum of **4** 15
- Mass Spectra of **4** 17
- UV-vis spectrum of **4** 18

Stability studies of complexes **3** and **4** in solution 19

X-ray Crystallography 21

Characterization of **2 (Ru-7-HSA)**

NMR spectra of **2**

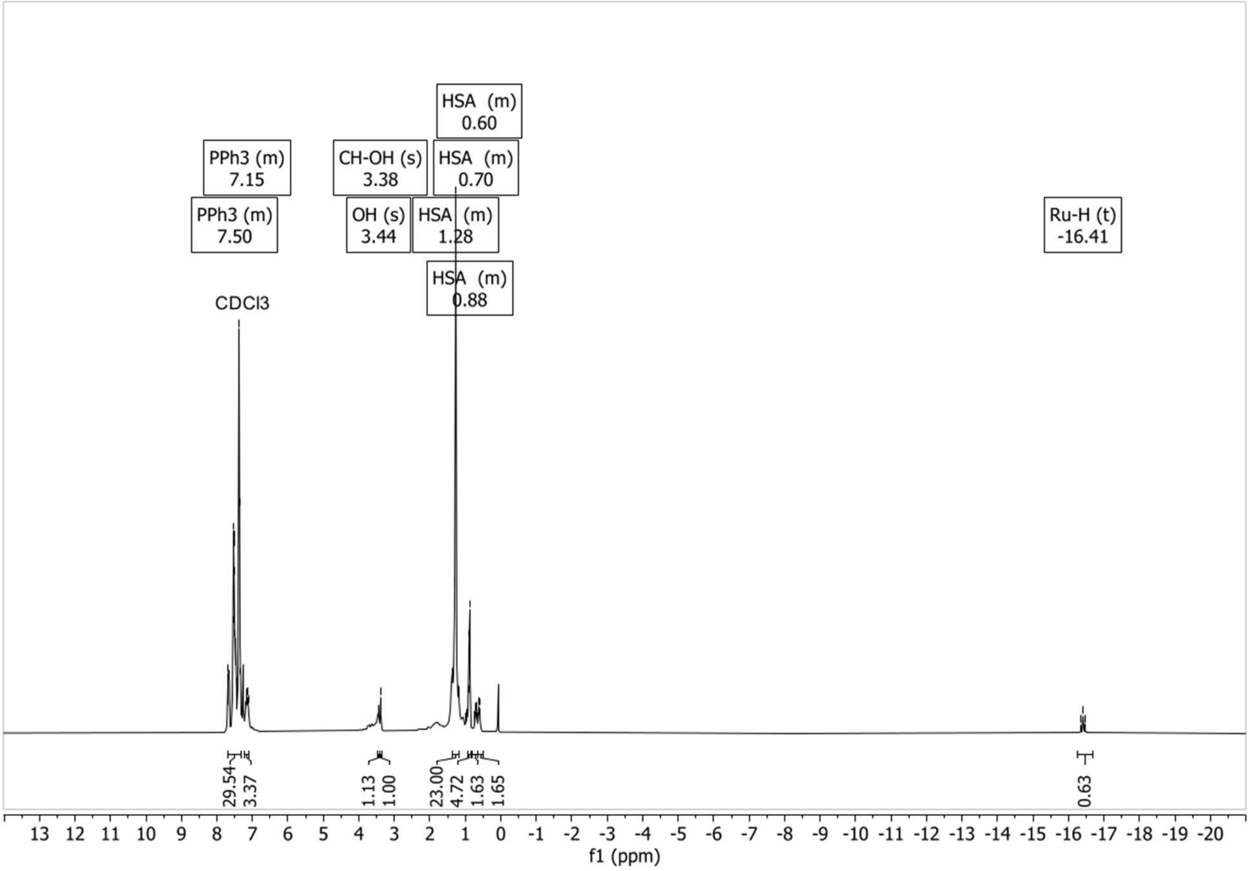

***S1**: ^{1}H NMR spectrum of **2** in $CDCl_3$*

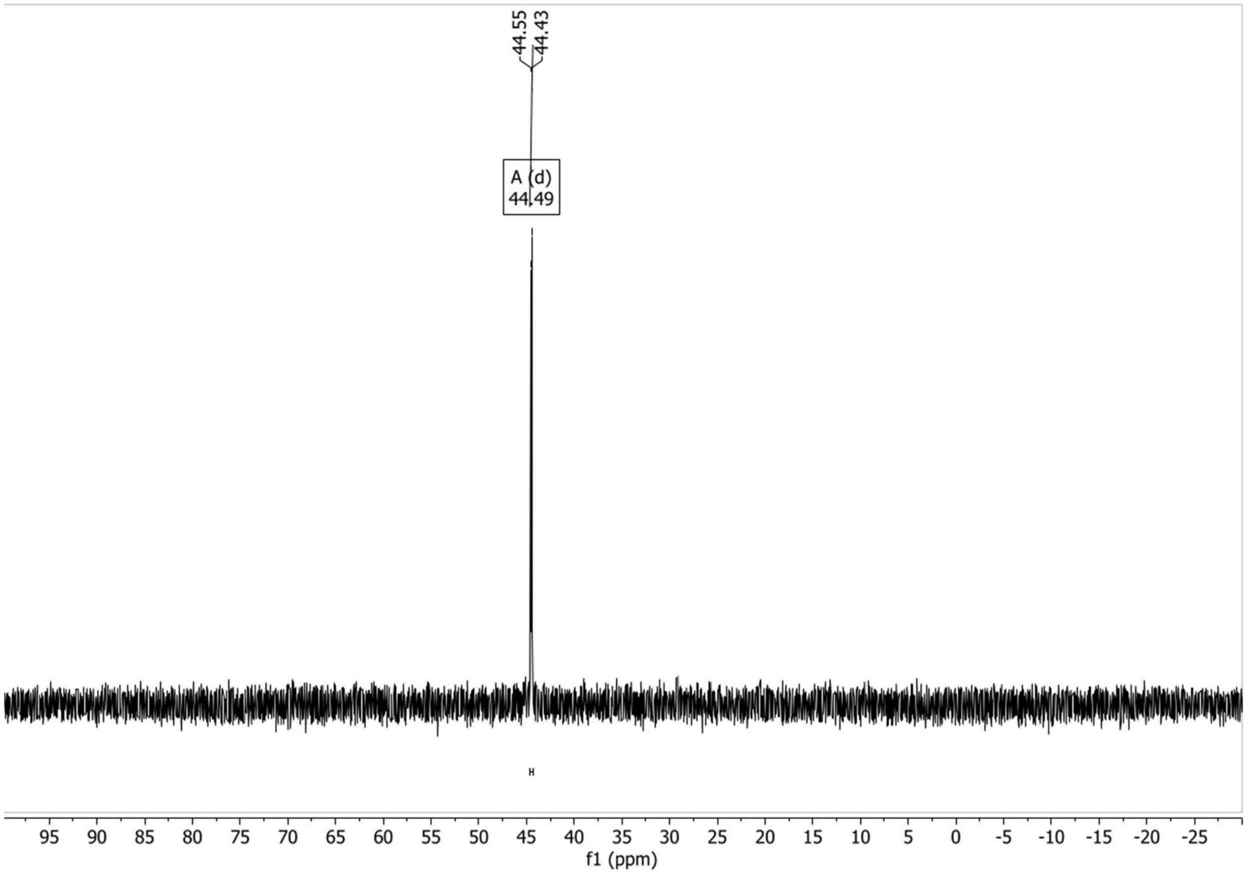

***S2**: ^{31}P spectrum of **2** in CDCl3*

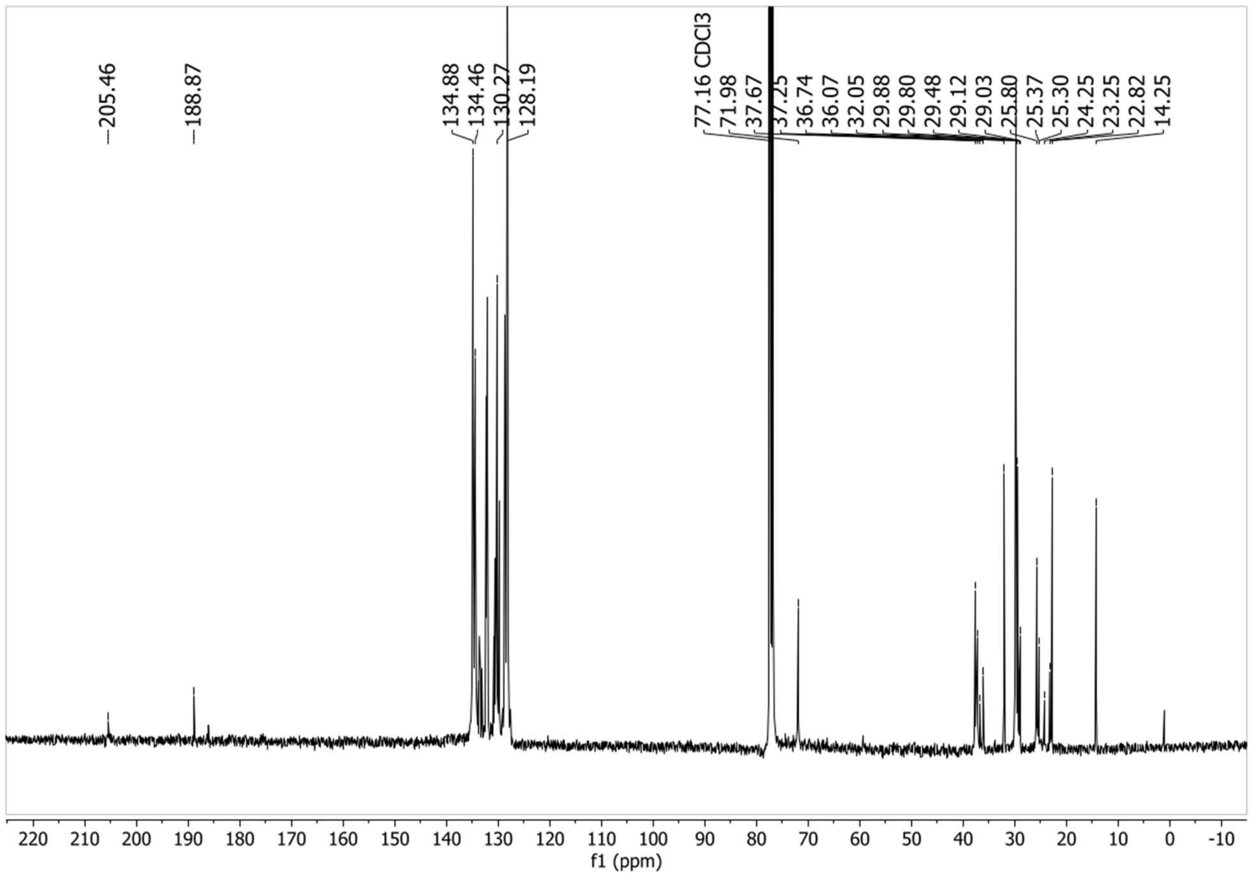

*S3: ^{13}C spectrum of **2** in $CDCl_3$*

IR spectrum of **2**

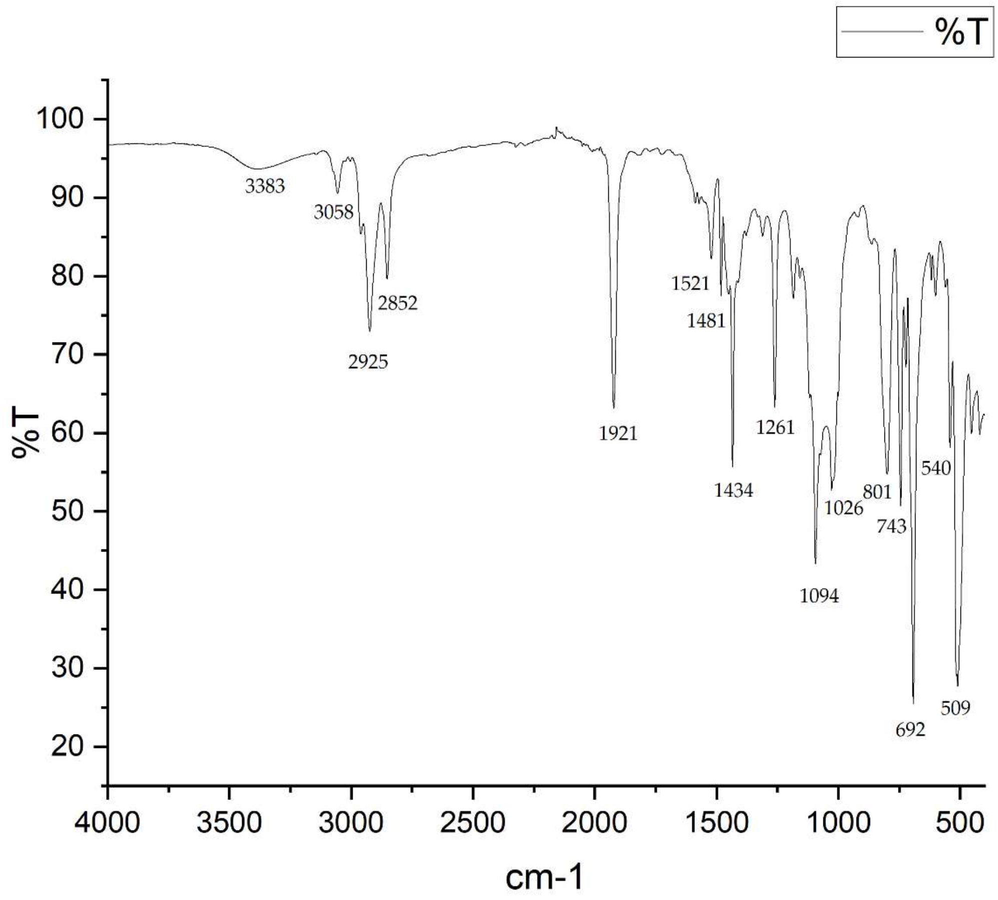

*S4: IR spectrum of **2***

Mass Spectra of **2**

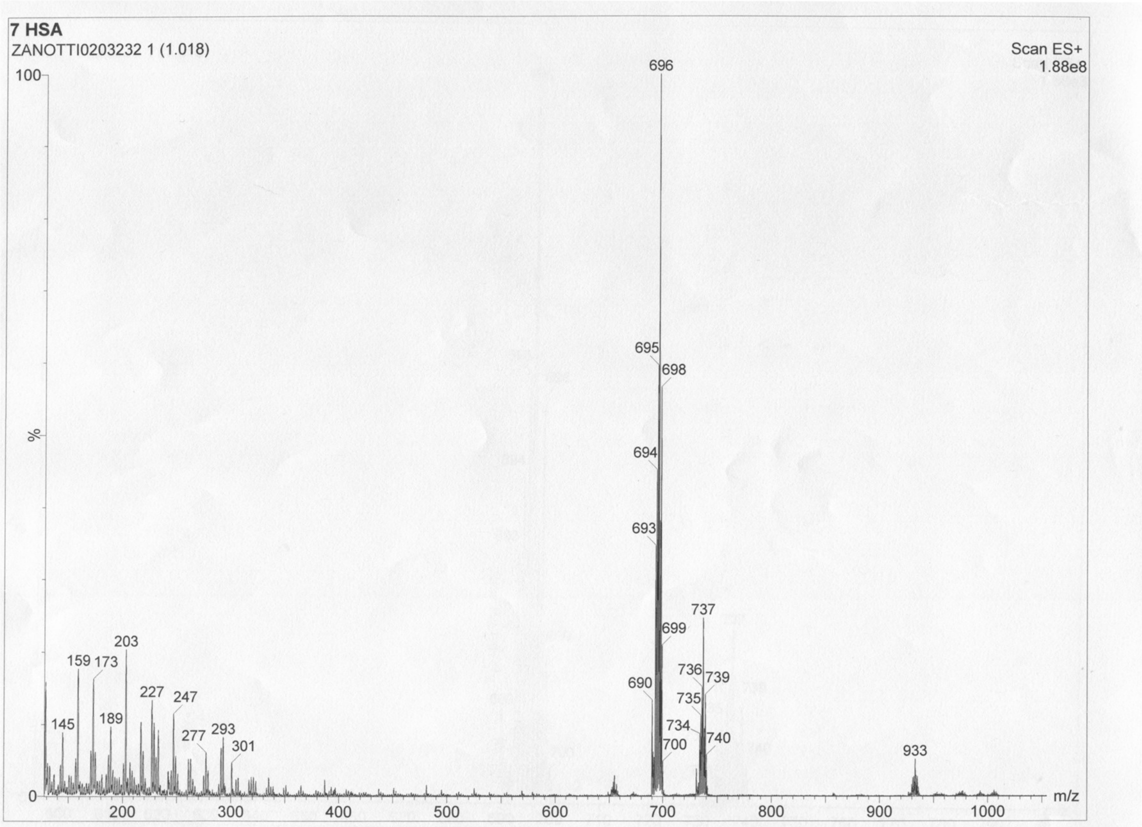

*S5: Mass Spectrum of **2** (Positive mode)*

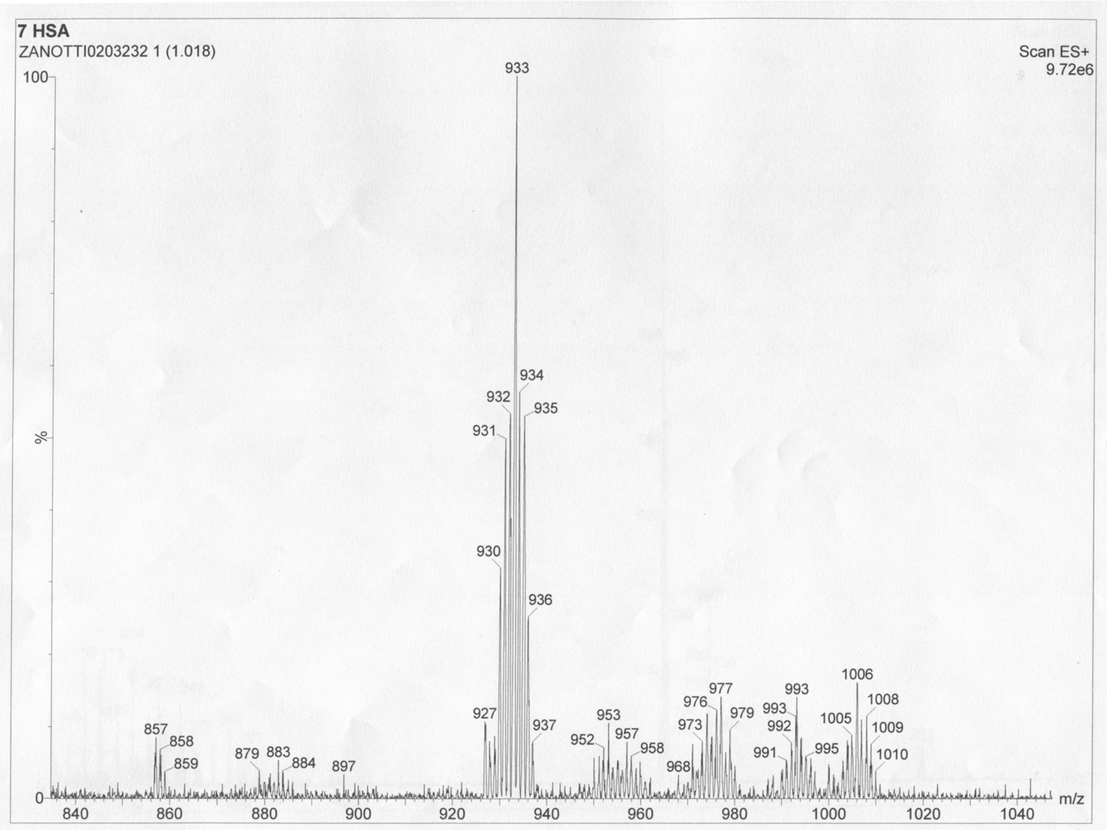

*S6: Mass spectrum of **2** (Positive mode, m/z: 840 - 1040)*

UV-vis spectrum of **2**

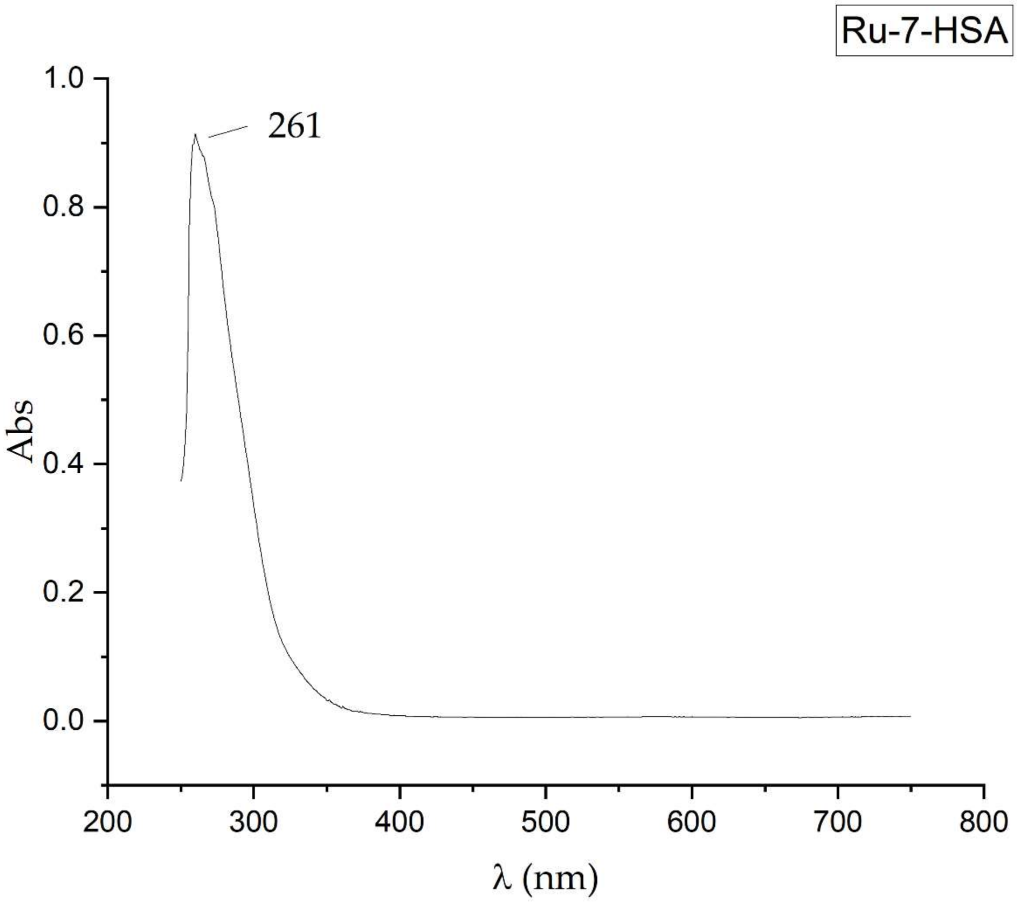

***S7**: UV-vis spectrum of **2** in DMSO*

Characterization of **3 (Ru-9-HSA)**

NMR spectra of **3**

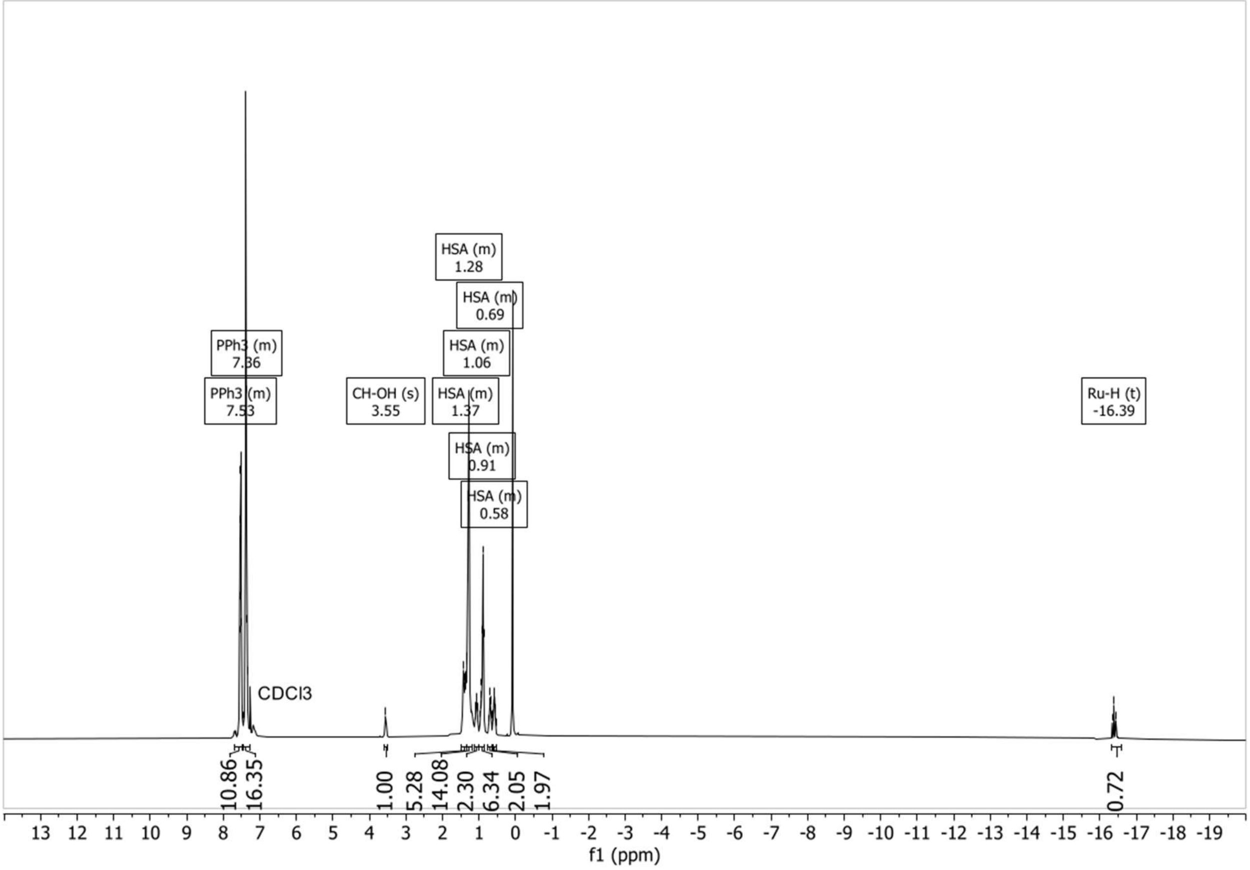

S8: *^{1}H NMR spectrum of **3** in $CDCl_3$*

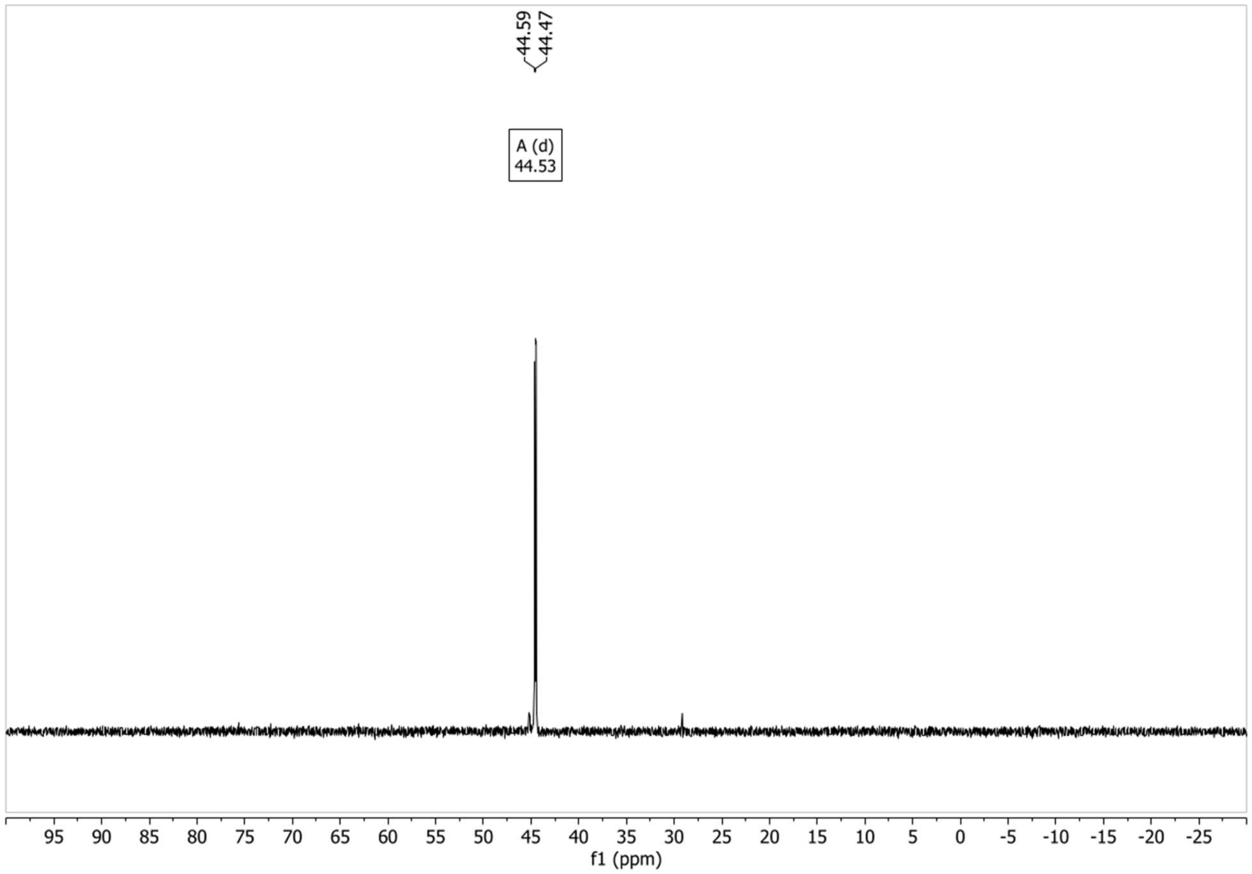

***S9**: ^{31}P NMR spectrum of **3** in $CDCl_3$*

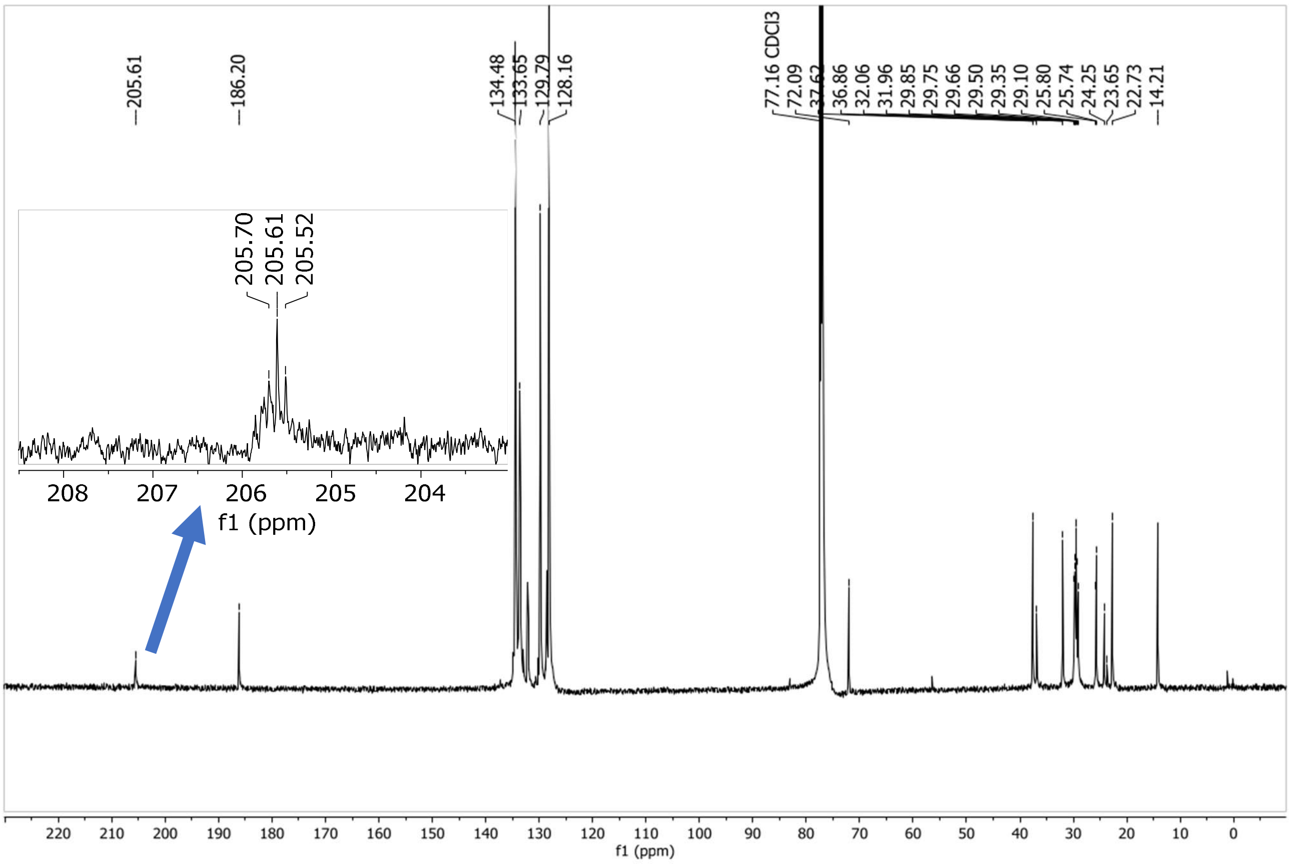

***S10**: ^{13}C spectrum of **3** in $CDCl_3$*

IR spectrum of **3**

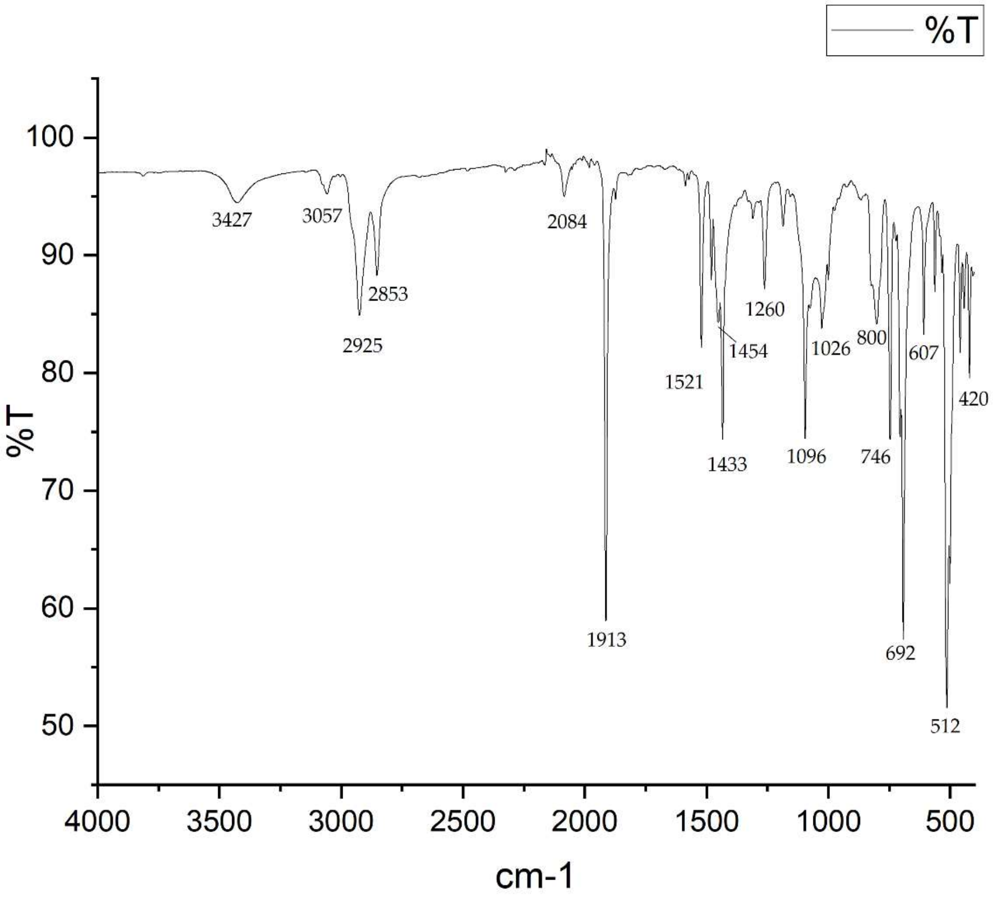

S11: IR spectrum of **3**

Mass Spectra of **3**

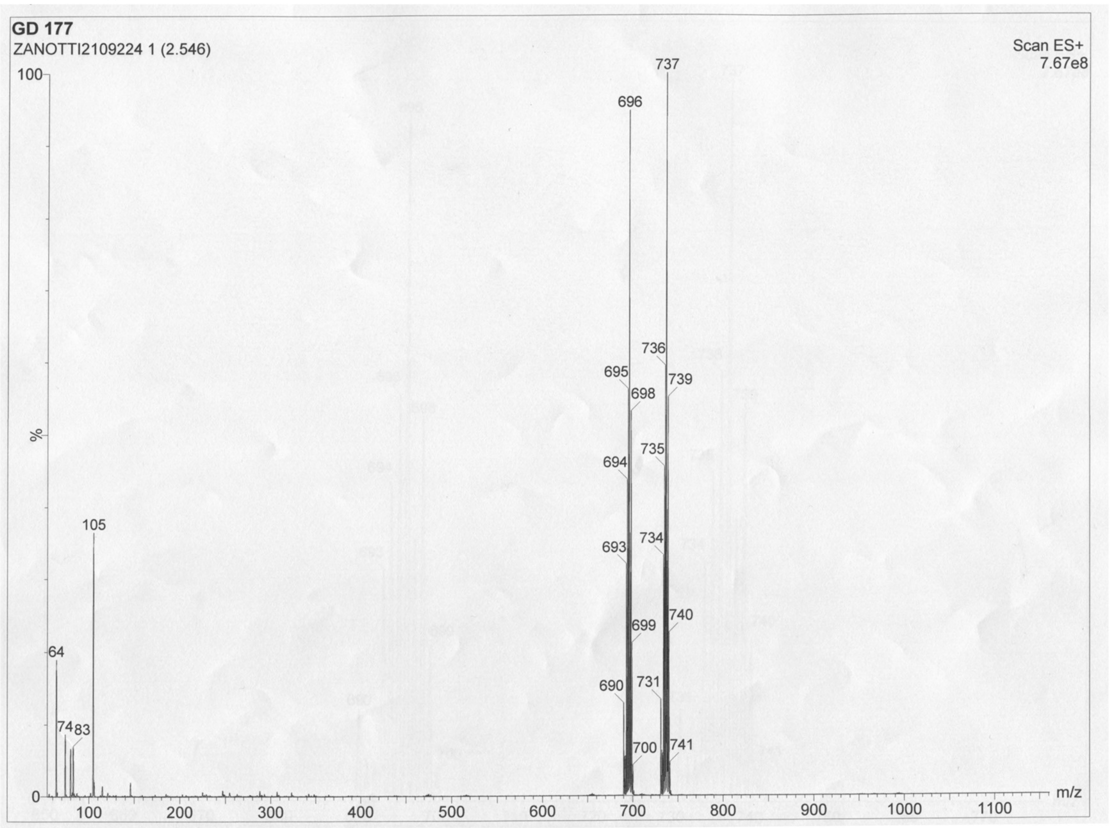

S12: *Mass spectrum of* ***3*** *(positive mode)*

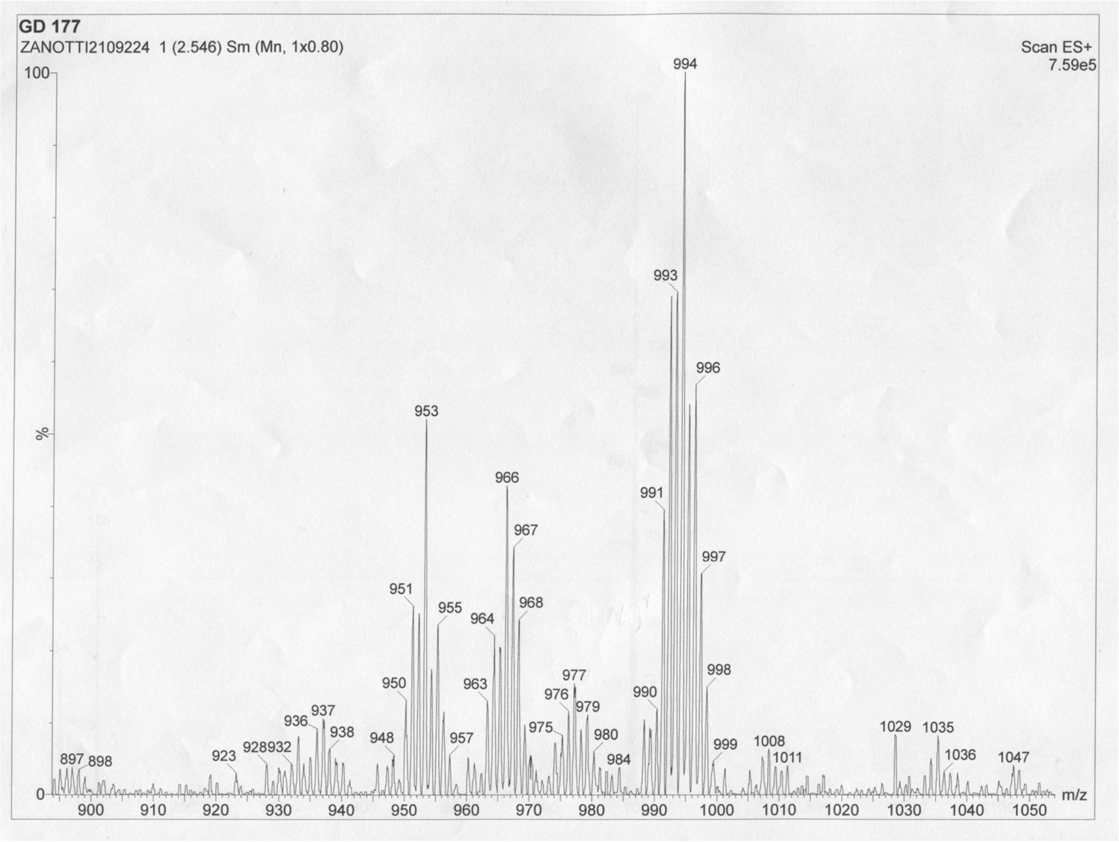

S13: *Mass spectrum of* ***3*** *(positive mode, m/z: 900 - 1050)*

UV-vis spectrum of **3**

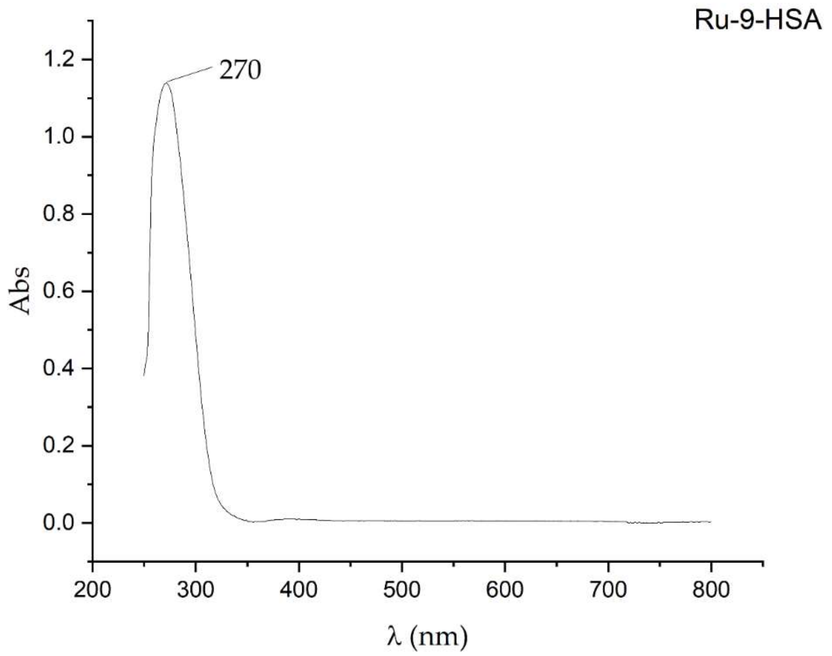

***S14**: UV-vis spectrum of **3** in DMSO*

Characterization of **4 (Ru-12-HSA)**

NMR spectra of **4**

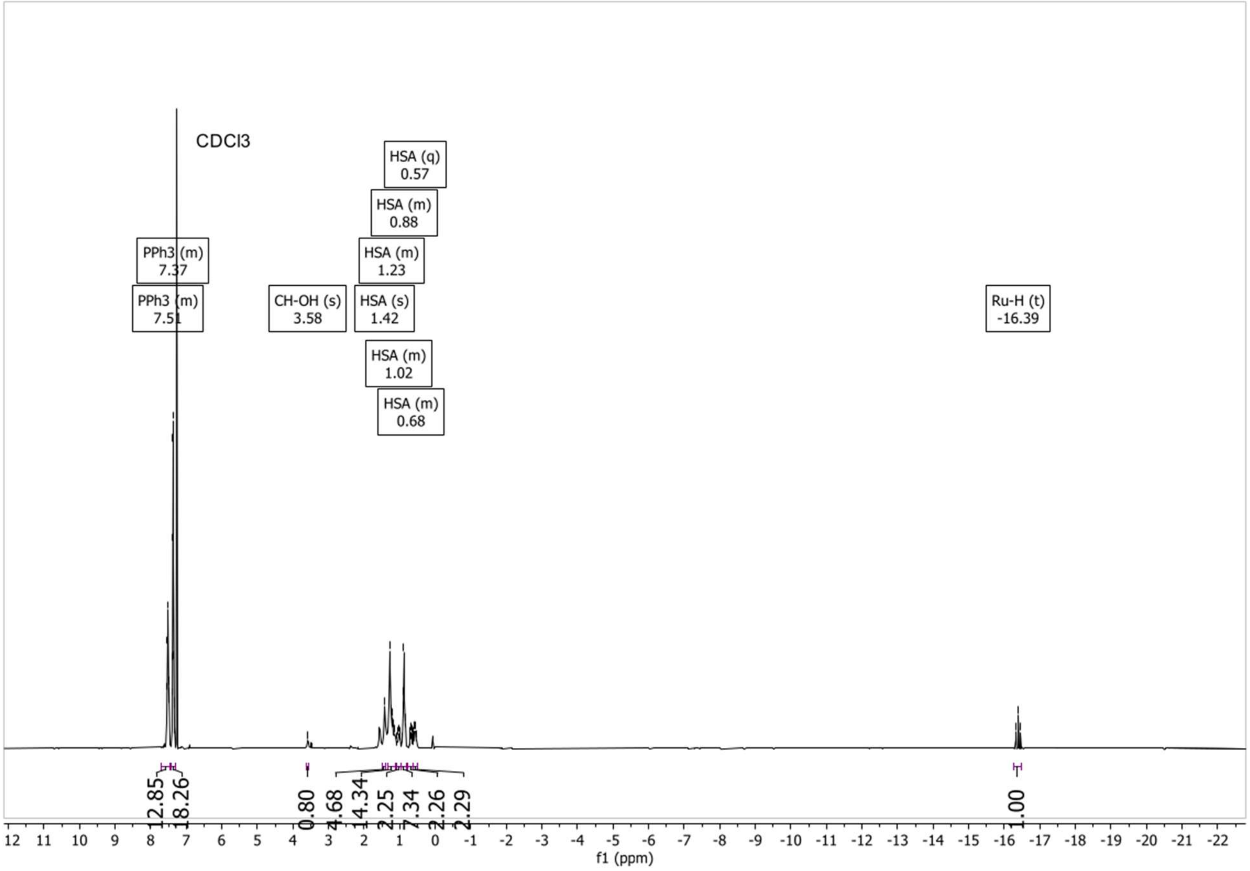

S15: *^{1}H NMR spectrum of **4** in CDCl*$_3$

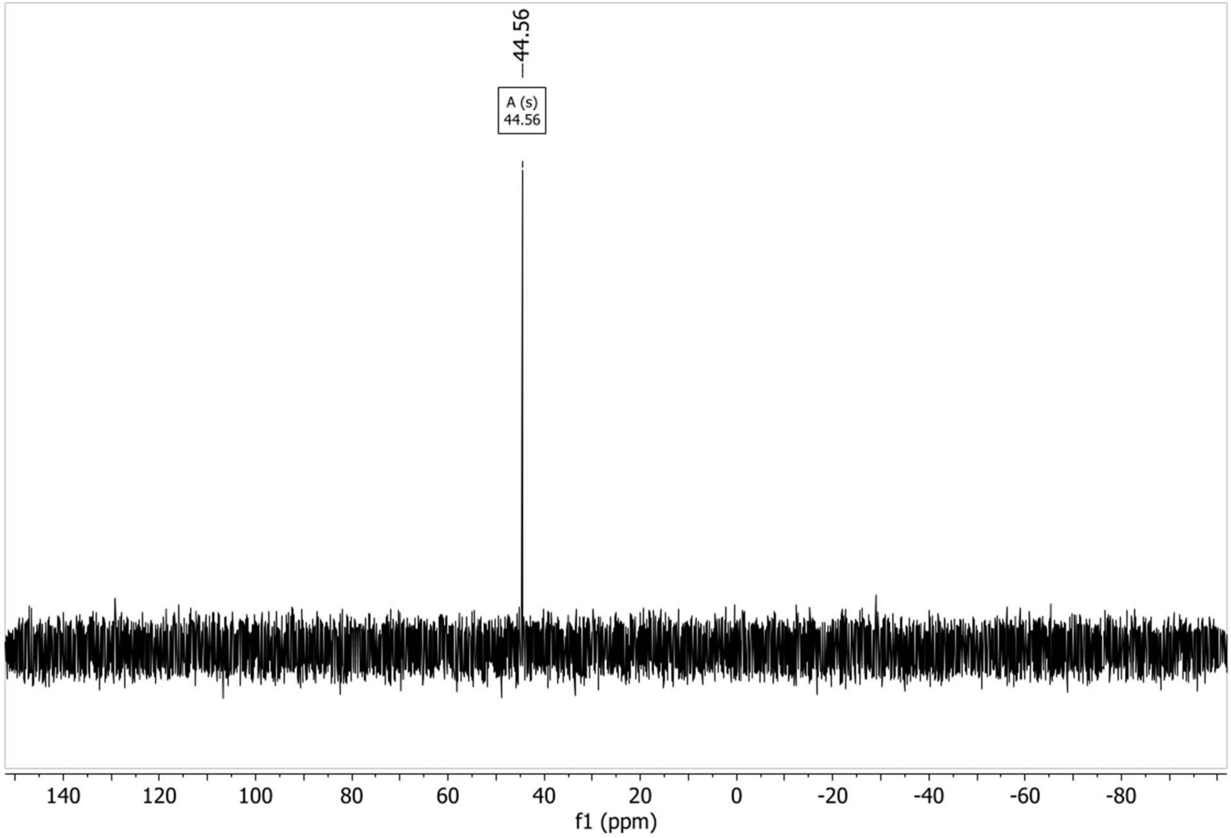

***S16**: ^{31}P NMR spectrum of **4** in $CDCl_3$*

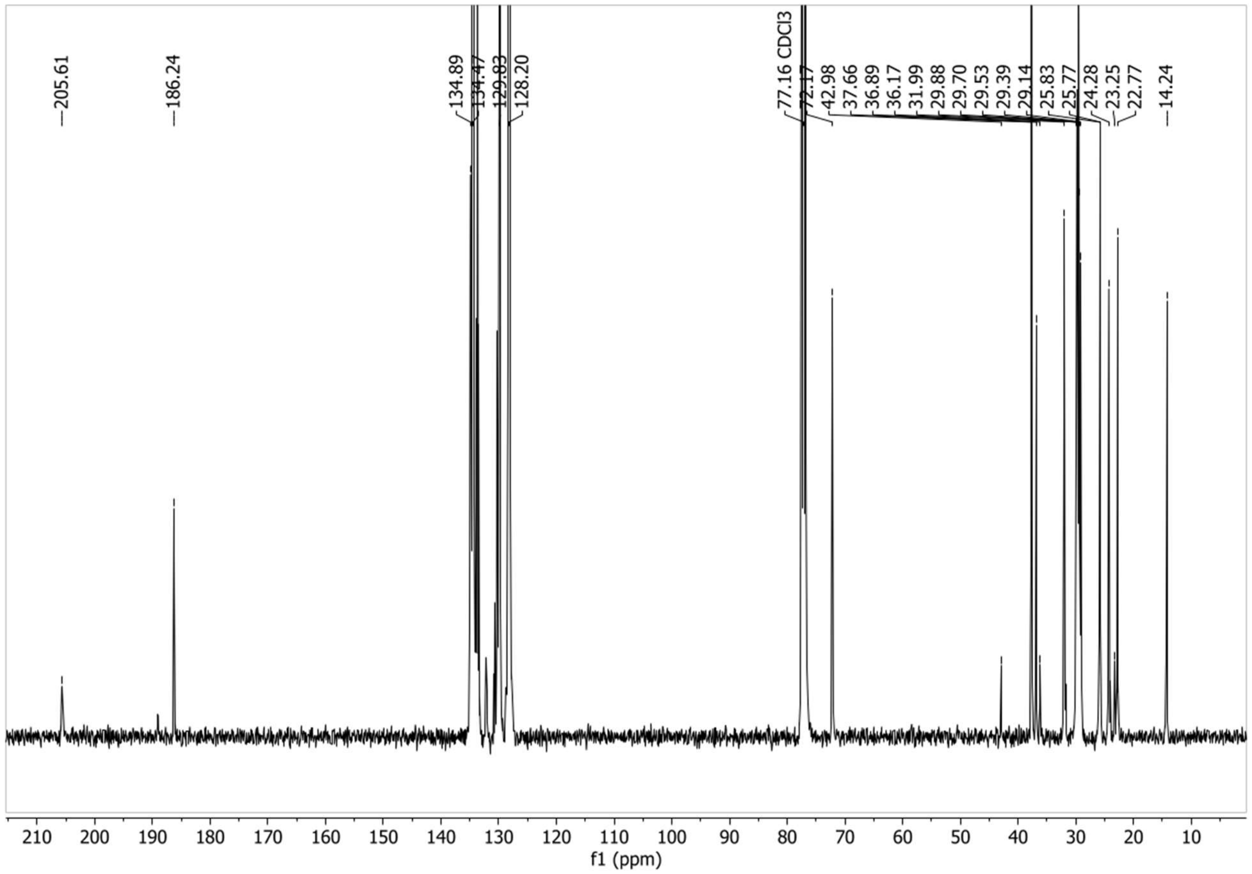

S17: *^{13}C NMR spectrum of **4** in $CDCl_3$*

IR Spectrum of **4**

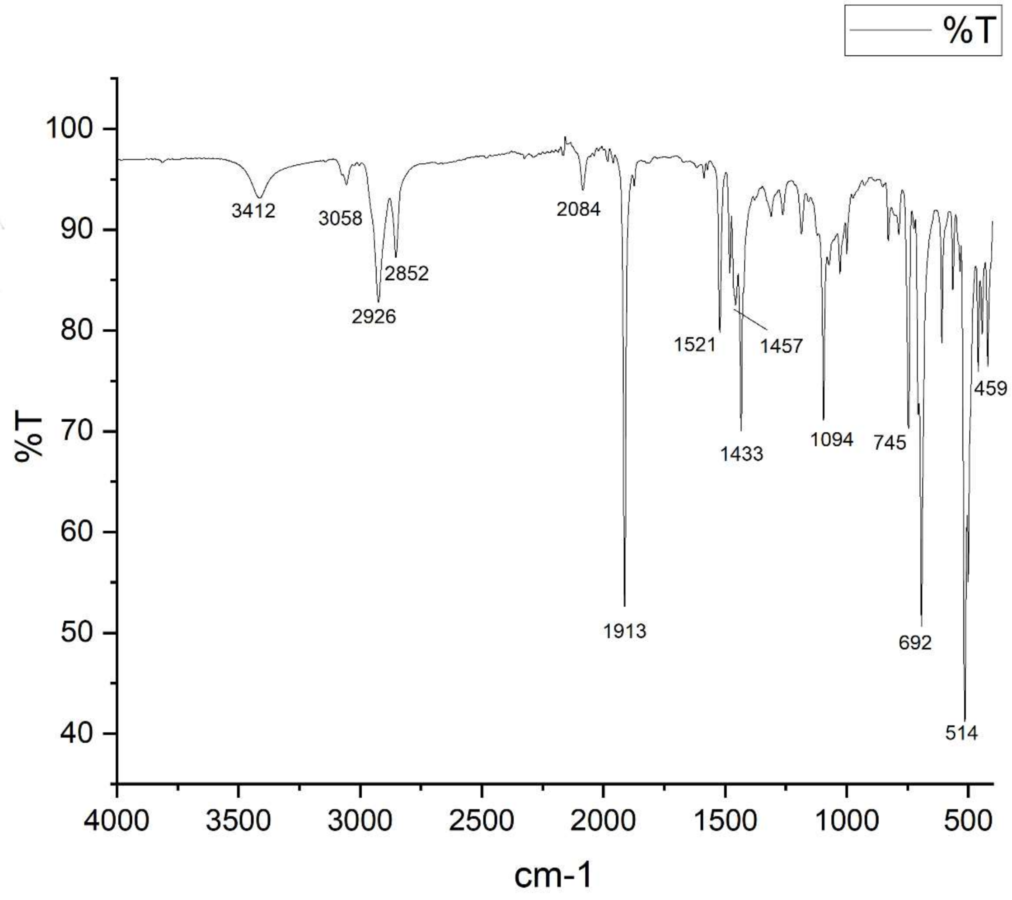

S18**: IR spectrum of **4

Mass Spectra of **4**

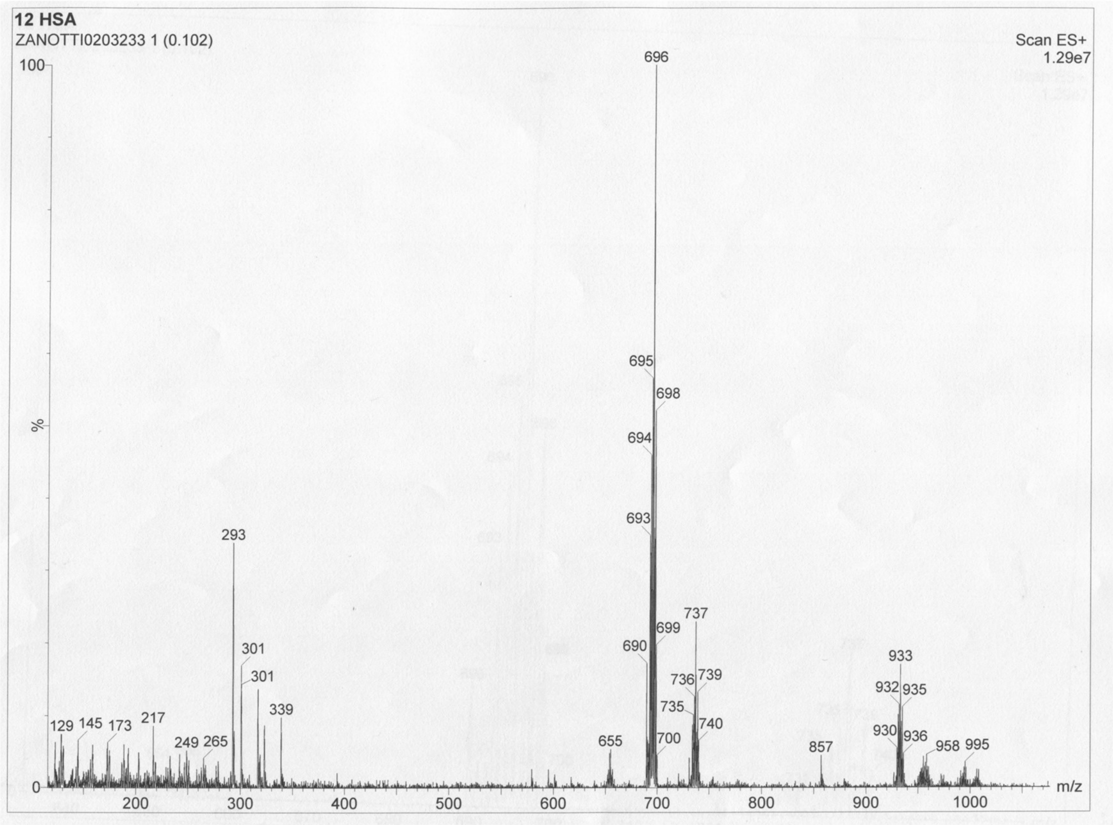

S19: *Mass spectrum of* ***4*** *(positive mode)*

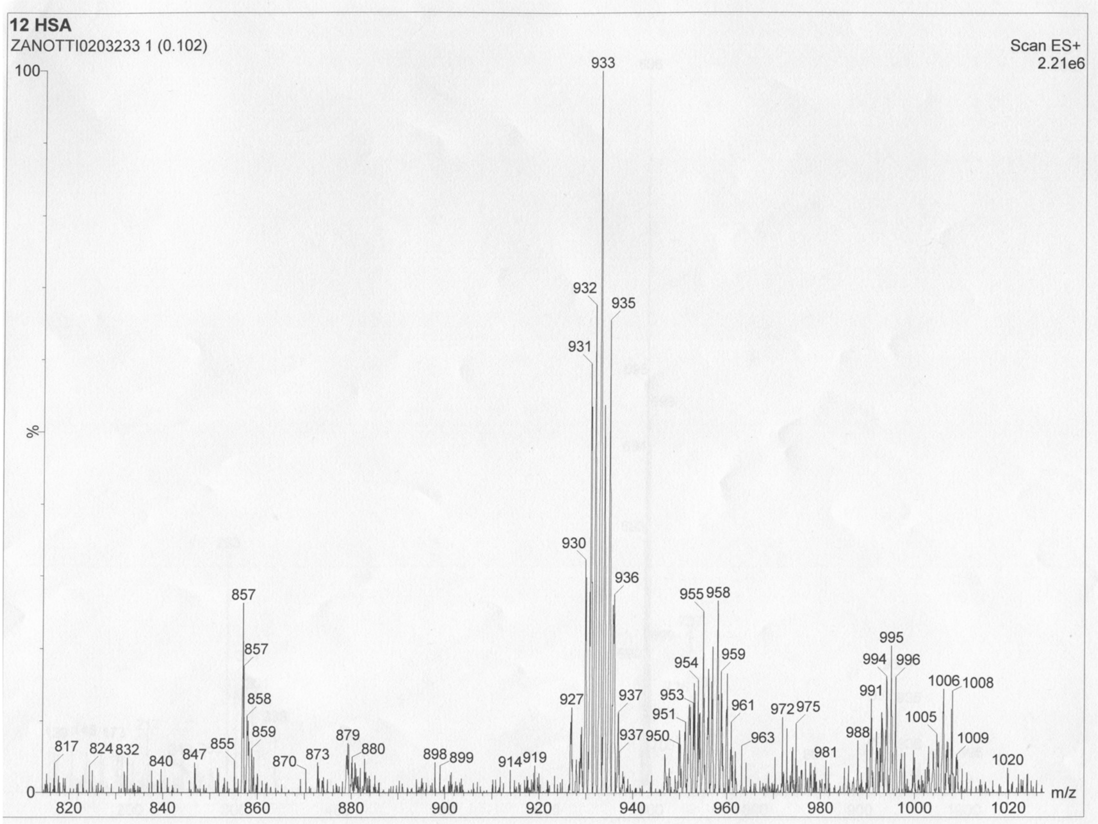

S20: *Mass spectrum of* ***4*** *(positive mode, m/z: 820 - 1020)*

UV-vis spectrum of **4**

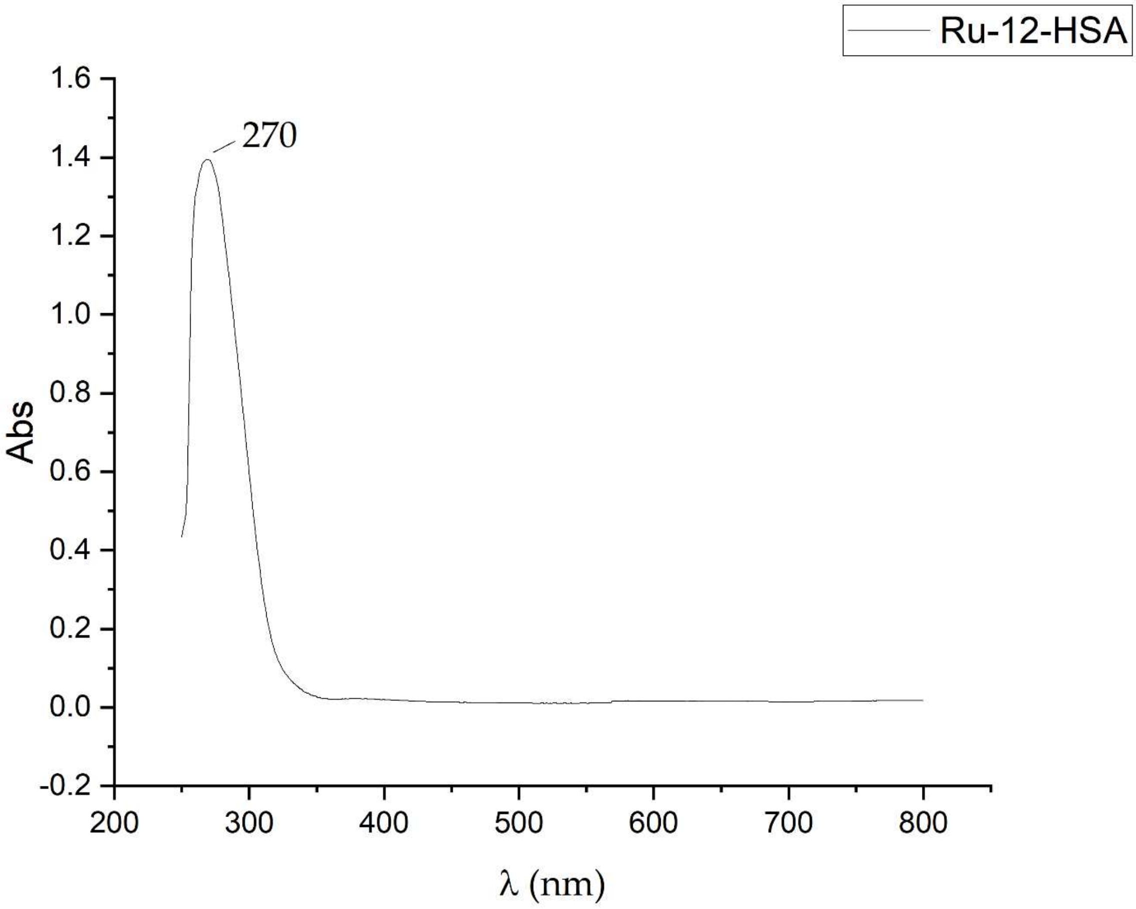

***S21**: UV-vis spectrum of **4** in DMSO*

Stability studies of complexes **3** and **4** in solution

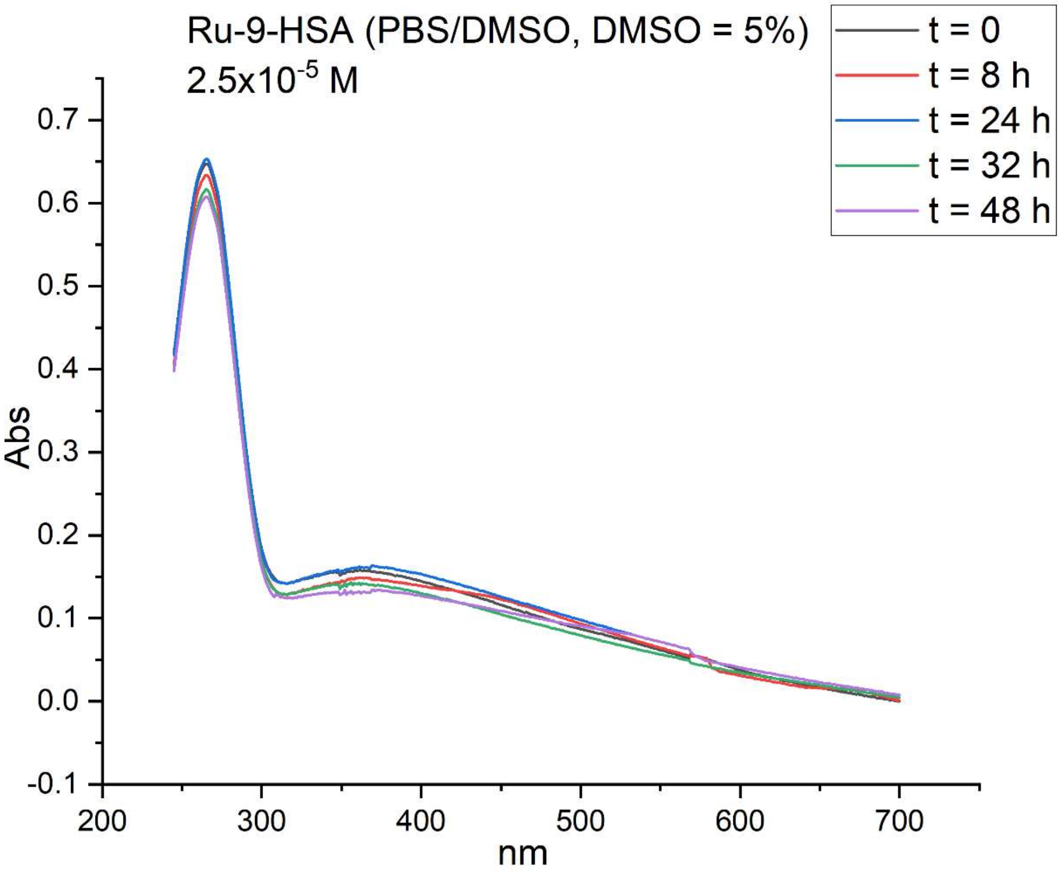

__S22__: Stability of __3__ in PBS/DMSO (DMSO = 5%) at 37°C over 48 h; time-resolved UV-vis spectrum.

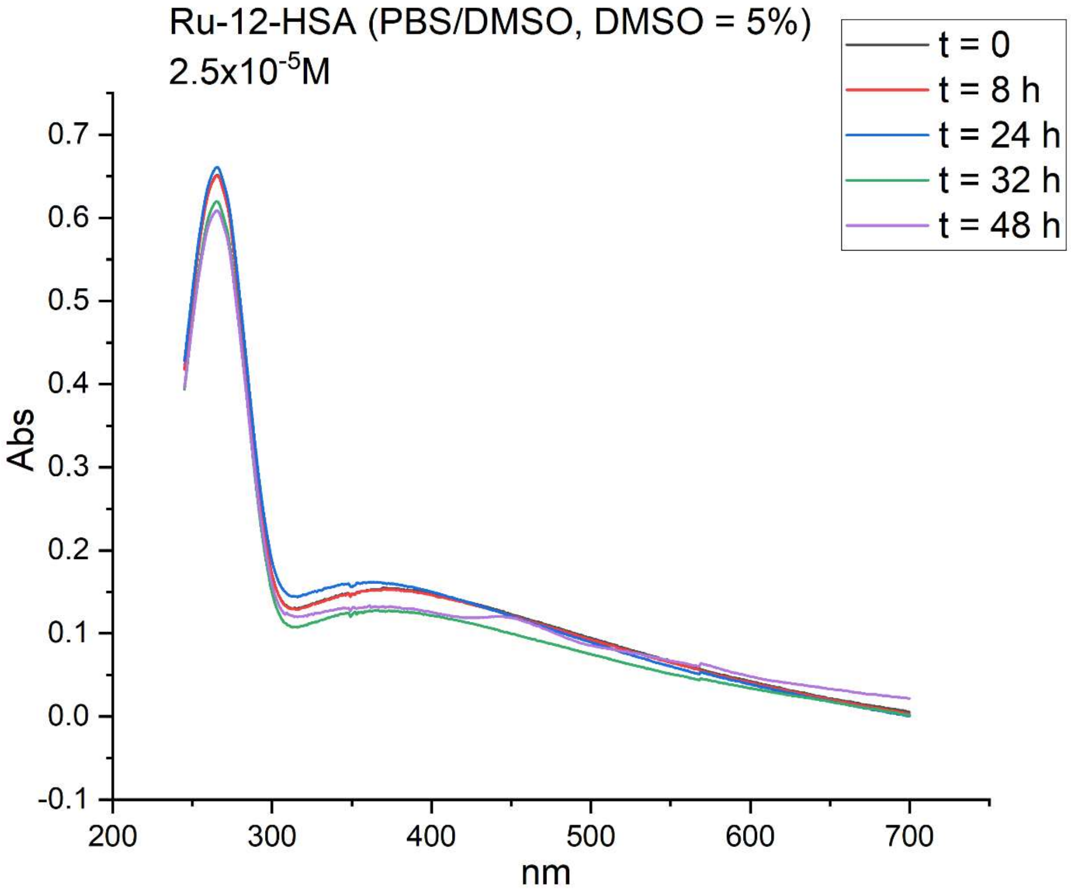

***S23**: Stability of **4** in PBS/DMSO (DMSO = 5%) at 37°C over 48 h; time-resolved UV-vis spectrum*

X-ray Crystallography

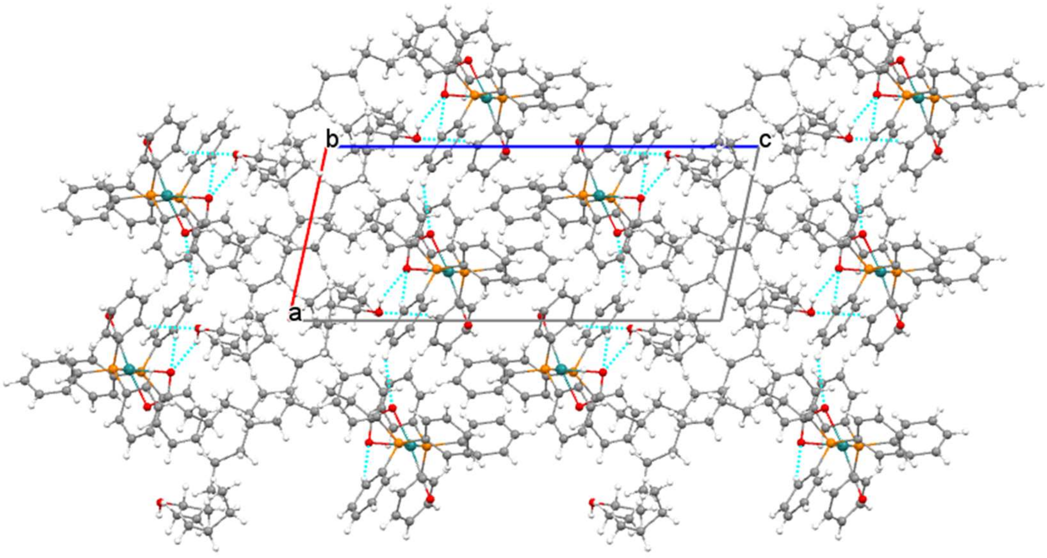

S24: View down the b axis of the crystal packing of 4

*Table S1. Crystal data and experimental details for **4***

Compound	**4**
Formula	$C_{55}H_{65}O_4P_2Ru$
Fw	953.08
T, K	100(2)
λ, Å	1.54178
Crystal symmetry	Triclinic
Space group	P-1
a, Å	9.9781(13)
b, Å	12.0328(15)
c, Å	22.364(3)
α	93.268(7)
β	99.999(7)
γ	113.041(6)
Cell volume, Å^3	2410.1(5)
Z	2
D_c, Mg m^{-3}	1.313
μ(Mo-K$_α$), mm^{-1}	3.607

F(000)	1002
Crystal size/ mm	0.10 x 0.04 x 0.04
θ limits, °	2.025 to 58.925
Reflections collected	31303
Unique obs. Reflections [$F_o > 4\sigma(F_o)$]	6848 [R(int) = 0.1227]
Goodness-of-fit-on F^2	1.041
R_1 (F)[a], wR_2 (F^2) [$I > 2\sigma(I)$][b]	0.1067, 0.2651
Largest diff. peak and hole, e. Å^{-3}	1.592 and -0.783

[a)] $R_1 = \Sigma||F_o|-|F_c||/\Sigma|F_o|$. [b] $wR_2 = [\Sigma w(F_o^2-F_c^2)^2/\Sigma w(F_o^2)^2]^{1/2}$ where $w = 1/[\sigma^2(F_o^2) + (aP)^2 + bP]$ where $P = (F_o^2 + F_c^2)/3$.

Table S2. *Intermolecular hydrogen bonds for* ***4*** *[Å and °].*

D-H...A	d(D-H)	d(H...A)	d(D...A)	<(DHA)
O3-H3…O1#1	0.84	2.10	2.74(2)	133(1)
C31-H31…O3#1	0.95	2.69	3.39(2)	131.2(8)
C47-H47…O2#2	0.95	2.71	3.43(2)	133

Symmetry transformations used to generate equivalent atoms:

#1 2-x, 1-y, -z; #2 x+1, z
